# Identification and validation of quantitative trait loci for chlorophyll content of flag leaf in wheat under different phosphorus treatments

**DOI:** 10.3389/fpls.2022.1019012

**Published:** 2022-11-17

**Authors:** Bin Yang, Nan Chen, Yifei Dang, Yuzhi Wang, Hongwei Wen, Jun Zheng, Xingwei Zheng, Jiajia Zhao, Jinxiu Lu, Ling Qiao

**Affiliations:** ^1^ Institute of Wheat Research, State Key Laboratory of Sustainable Dryland Agriculture, Shanxi Agricultural University, Linfen, China; ^2^ College of Agronomy, Shanxi Agricultural University, Taiyuan, China

**Keywords:** wheat, phosphorus deficiency, chlorophyll, quantitative trait locus, validation

## Abstract

In wheat, the leaf chlorophyll content in flag leaves is closely related to the degree of phosphorus stress. Identifying major genes/loci associated with chlorophyll content in flag leaves under different phosphorus conditions is critical for breeding wheat varieties resistant to low phosphorus (P). Under normal, medium, and low phosphorus conditions, the chlorophyll content of flag leaves was investigated by a double haploid (DH) population derived from a cross between two popular wheat varieties Jinmai 47 and Jinmai 84, at different grain filling stages. Chlorophyll content of the DH population and parents decreased gradually during the S1 to the S3 stages and rapidly at the S4 stage. At the S4 stage, the chlorophyll content of the DH population under low phosphorus conditions was significantly lower than under normal phosphate conditions. Using a wheat 15K single-nucleotide polymorphism (SNP) panel, a total of 157 QTLs were found to be associated with chlorophyll content in flag leaf and were identified under three phosphorus conditions. The phenotypic variation explained (PVE) ranged from 3.07 to 31.66%. Under three different phosphorus conditions, 36, 30, and 48 QTLs for chlorophyll content were identified, respectively. Six major QTLs *Qchl.saw-2B.1*, *Qchl.saw-3B.1*, *Qchl.saw-4D.1*, *Qchl.saw-4D.2*, *Qchl.saw-5A.9* and *Qchl.saw-6A.4* could be detected under multiple phosphorus conditions in which *Qchl.saw-4D.1*, *Qchl.saw-4D.2*, and *Qchl.saw-6A.4* were revealed to be novel major QTLs. Moreover, the closely linked SNP markers of *Qchl.saw-4D.1* and *Qchl.saw-4D.2* were validated as KASP markers in a DH population sharing the common parent Jinmai 84, showed extreme significance (*P <*0.01) in more than three environments under different phosphorus conditions, which has the potential to be utilized in molecular marker-assisted breeding for low phosphorus tolerance in wheat.

## Introduction

Wheat (*Triticum aestivum* L.) is a hexaploid (2n=6x=42) staple food crop, feeding approximately 30% of the world’s population (Mayer et al., 2014), and estimated that wheat production will need to increase by 60% to meet human demands (Langridge, 2013). Thus, increasing the potential yield of wheat is the primary aim of breeders.

Flag leaf is one of the most crucial photosynthetic organs during the grain filling stage in wheat, contributing up to fifty percent of photosynthate to yield formation (Araus and Tapia, 1987). The chlorophyll of flag leaf is the main element to participate in photosynthesis in wheat. Chlorophyll content is significantly and positively correlated with photosynthetic efficiency (Avenson et al., 2005), which directly influences the accumulation of photosynthetic assimilates and yield formation (Jiang et al., 2004). The chlorophyll content is highly susceptible to degradation under abiotic stress conditions such as drought, high temperature, intense light, and nutritional deficiency, resulting in early leaf death and crop yields loss (Benbella and Paulsen, 1998; Fang et al., 2004; Li et al., 2010; Li et al., 2014; Talukder et al., 2014; Li et al., 2015). Under stress conditions, stress-tolerant genotypes usually display higher chlorophyll content and thus maintain stronger photosynthetic efficiency, which helps to delay leaf senescence (Kumar et al., 2012), prolong photosynthetic time (Farooq et al., 2014), and subsequently contribute to the formation of yield (Bogard et al., 2011). Therefore, investigating the molecular genetic mechanism of regulating chlorophyll content in flag leaves is crucial for improving wheat production potential under stressed conditions.

The chlorophyll content is a complex quantitative character regulated by both genotype and environment (Zhang et al., 2009). Mapping and cloning genes controlling chlorophyll are essential for studying the genetic characteristics of this trait (Thomas and Ougham, 2014; Rasheed et al., 2020). With increased research on chlorophyll-related traits in recent years, over 900 QTLs for chlorophyll content have been identified in rice, and at least eight stay-green related genes, including *DYE1*, *SGR*, and *EF8*, have been cloned (Morita et al., 2009; Feng et al., 2014; Yamatani et al., 2018; Yang et al., 2022). Research on the genetic mechanism that regulates chlorophyll in wheat lags behind model crops such as rice (Gupta et al., 2020). Under drought, high temperature, light intensity, and nutrient stress, QTL analysis of chlorophyll content has been performed (Fang et al., 2004; Li et al., 2010; Vijayalakshmi et al., 2010; Christopher et al., 2018; Maulana et al., 2018; Kamal et al., 2019), and hundreds of QTLs were identified on 21 chromosomes of wheat (Kumar et al., 2010; Li et al., 2010; Vijayalakshmi et al., 2010; Zhang et al., 2010; Kumar et al., 2012; Ilyas et al., 2014; Saleh et al., 2014; Barakat et al., 2015; Yang et al., 2016a; Yang et al., 2016b; Shi et al., 2017; Christopher et al., 2018; Puttamadanayaka et al., 2020; Yan et al., 2020; Yang et al., 2022). However, most studies used low-density SSR markers and were only identified in a few years/environments. The QTLs have not been confirmed for many years/environments, and most of them are minor QTLs that are easily affected by the environment, and this makes it challenging to use molecular markers to assist in breeding.

Single nucleotide polymorphism (SNP) mainly refers to DNA sequence polymorphisms caused by single nucleotide changes and small insertions/deletions (Useche et al., 2001; Tang et al., 2006). Compared to SSR markers, SNP markers have high throughput (Collard and Mackill, 2008), high abundance, and relative stability. They have been widely used in high-density genetic linkage map construction, fine mapping, functional marker development, and marker-assisted breeding (Zhang et al., 2013; Qi et al., 2014; Wang et al., 2019a). Several major stay-green QTLs/genes in wheat have been fine-mapped with the SNP array. The gene *els1* has been mapped in the WGGB303-WGGB305 marker interval on 2BS, with a genetic distance of 1.5 cM (Li et al., 2018), while the *els2* gene mapped to the 2BIP09-2BIP14 marker interval on 2BL, with a genetic distance of 0.95 cM (Wang et al., 2020). Ren et al. (2022) reported that two major stay-green QTLs, *QSg.sau-2B.1* and *QSg.sau-6A.2*, were located on chromosomes 2B and 6A, respectively. However, the number of major and stable QTLs/genes identified in multiple environments remains limited. There is no report on gene map-based cloning that regulation wheat chlorophyll. Thus, Gupta et al. (2020) consider it necessary to apply SNP markers to identify more major QTLs controlling stay-green related traits in multiple environments, and these would establish the foundation for map-based cloning.

The leaf chlorophyll content is strongly connected to phosphorus stress (Ma et al., 2013; Maharajan et al., 2018). Phosphorus is one of the essential nutrients for wheat’s growth, development, and yield production. Phosphorus deficit is one of the most significant abiotic factors affecting wheat yield formation in China and worldwide. QTL analysis for chlorophyll content under phosphorus deficit conditions has been performed in rice, corn, soybean, and other crops (Kanbe et al., 2008; Cai et al., 2012; Li et al., 2016) but not in wheat. Since the genes that control chlorophyll content change during the filling period (Atchley and Zhu, 1997), genetic analysis of chlorophyll content at different filling periods is sure to help find more genetic information (Yang et al., 2016b). Therefore, QTL analysis of chlorophyll content at different filling stages under different phosphorus conditions is necessary to detect more stable and major QTL.

In this study, the DH1 population (Jinmai 47 × Jinmai 84) was used to analyze the QTL of chlorophyll content of flag leaves at different filling stages under normal phosphorus (CK), medium phosphorus (MP), and low phosphorus (LP) conditions, and the DH2 population (Jinmai 919 × Jinmai 84) was used to validate the major QTL. Our goals for this study are 1) to find out how different levels of phosphorus influence the dynamic changes in chlorophyll content in flag leaves at the grain filling stages; 2) to detect the major QTL which regulates chlorophyll content in flag leaves at different stages of grain filling; and 3) to recognize how different levels of phosphorus affect QTLs related to chlorophyll content in order to develop molecular markers for low-phosphorus tolerance-assisted breeding.

## Materials and methods

### Plant materials and plot design

**Figure 1:**
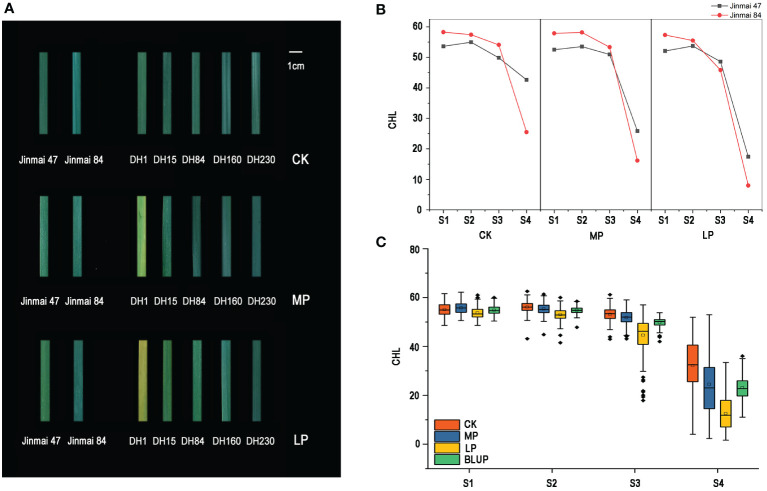
Leaf phenotype of parents and lines of DH1 population **(A)**. Each leaf intercepts an equal area. Changes in chlorophyll content of parents (Jinmai 47 and Jinmai 84) under normal (CK), medium (MP), and low phosphorus (LP) conditions at four stages **(B)**. Box diagram of chlorophyll content change in DH1 populations under three phosphorus conditions at four stages after flowering **(C)**.

The DH1 population was constructed by the crossing between Jinmai 47 × Jinmai 84, with a total of 201 lines, in which the female parent Jinmai 47 is a dryland variety, with light green leaves and high-stress resistance, and the male parent Jinmai 84 is a high-yield wetland variety with dark green leaves chosen by Cotton Research Institute of Shanxi Academy of Agricultural Sciences (**Figure 1A**). Whereas, the DH2 population was constructed by crossing between Jinmai 919 × Jinmai 84, a total of 160 lines, in which Jinmai 919 is a dryland variety approved by the Wheat Research Institute of Shanxi Agricultural University in 2018 with strong drought resistance. The DH1 and DH2 populations were planted in Hongbao Experimental Station, Linfen, China, in 2020-2021, including six experimental sites (normal phosphorus, CK1, and CK2; medium phosphorus, MP1, and MP2; low phosphorus, LP1, and LP2). Urea (CO(NH_2_)_2_) 168.75 kg·ha^-1^ and potassium chloride (KCl) 49.17 kg·ha^-1^ fertilizers were applied to all six experimental sites. As well as phosphorus pentaoxide (P_2_O_5_) 196 kg·ha^-1^ was also applied to CK1 and CK2, P_2_O_5_ 84 kg·ha^-1^ was applied to MP1 and MP2, and LP1 and LP2 without phosphate fertilizer. The data of nutritional analysis of the soil are shown in **Table S1**. Treatment of DH2 population fertilization was consistent with DH1. The field design for both populations consisted of randomized complete blocks with three replications. Each plot consisted of two 1.5 m rows spaced 0.3 m apart at 21 seeds per row. After sowing, CK2, MP2, and LP2 were irrigated before the overwintering, jointing, and flowering, respectively. Each irrigation amount of 700 m^3^·hm^-2^. CK1, MP1, and LP1 relied on natural precipitation during the whole growth period. The precipitation for the wheat growth period in 2020-2021 was around 147 mm, and the precipitation after flowering is shown in **Figure S1**.

### Evaluation and measurement of phenotypic traits

Ten plants were randomly selected and marked from the parents and each line of the DH1 population at the flowering stage based on similar flowering, development, and growth. The chlorophyll content of flag leaf was measured by SPAD-502 Plus chlorophyll meter (Konica-Minolta, Japan) from 7:00 to 10:00 hours at 7 days (S1), 14 days (S2), 21 days (S3), and 28 days (S4) after flowering (Yang et al., 2016a). Each flag leaf was measured three times at the base, middle, and tip, of leaves, and the average value was used for analysis. The determination method for DH2 population was the same as that for DH1 population, and the chlorophyll content of flag leaves was only measured at ten days after anthesis.

### Data analysis

The analysis of variance (ANOVA), correlation analysis, and the Student’s *t*-test (*P <*0.05) of measured phenotype values in different environments were performed by SPSS 21.0 software (SPSS, Chicago, IL, USA; http://www.spss.com) (Yang et al., 2022). SAS (SAS Institute, Cary, NC, USA; https://www.sas.com) was used for calculating the best linear unbiased prediction (BLUP) and broad sense heritability (*H* ^2^) for chlorophyll content of flag leaves in different environments (Smith et al., 1998). The low phosphorus tolerance coefficient (LPTC) is calculated according to the method of Zhang et al. (2011). The LPTC was calculated as MP (LP)/CK, where MP (LP) was the chlorophyll content measured under medium phosphorus (low phosphorus) conditions, and CK was the value obtained under normal phosphorus environments.

### Construction of genetic linkage map and QTL mapping

DNA was extracted from all DH lines and respective parents using the CTAB method (Vijayalakshmi et al., 2010). The DH1 population was genotyped with a 15K SNP panel (Qiao et al., 2022), which yielded 14,868 SNPs and used in the linkage analysis *via* QTL IciMapping 4.1 (Meng et al., 2015) and visualized with JoinMap 4.0. The QTL was detected using WinQTLCart version 2.5 (https://brcwebportal.cos.ncsu.edu/qtlcart/WQTLCart.htm) based on the composite interval mapping method. QTLs were claimed to be significantly above the 2.5 logarithms of odds (LOD) threshold. The QTL contributing more than 10% to phenotypic variation in a certain environment (including BLUP) and detected in three environments (including BLUP) was considered a stable and major QTL. QTLs less than 1 cM apart or sharing common flanking markers were treated as a single locus and named according to McCouch et al. (1997).

### Development of markers and validation of major QTLs

To develop kompetitive allele specific PCR (KASP) markers from peak marker of the major QTLs, two SNP specific primers (F1/F2) and one common primer (R) were designed for each KASP marker. The F1 tail added specific sequences that could bind to FAM fluorescence, and the F2 tail added specific sequences that could bind to HEX fluorescence. KASP primers were designed by Polymarker (http://www.polymarker.info/) and synthesized by Beijing Jiacheng Biotechnology Co., Ltd (**Table S2**). The developed KASP markers were used in DH2 population for validation. Following genotyping, the validation population was divided into two groups, and *t*-tests assessed differences in chlorophyll content of flag leaves between groups in SAS V8.0 (Yang et al., 2022).

### Identification of candidate genes

The genes in the target region of QTL were identified by using the JBrowse platform of the Triticeae Multi-omics website (http://wheatomics.sdau.edu.cn). The GO (Gene ontology) database was used for functional annotation and enrichment analysis of genes in these regions based on the BioMart-Martview database (http://plants.ensembl.org/biomart/martview/), and genes associated with chlorophyll and phosphorus were selected. The expression of related genes was obtained using the expVIP website (http://www.wheat-expression.com/). The differential expression genes related to phosphorus stress were further analyzed.

## Results

### Phenotypic variations and correlations of chlorophyll content under different phosphorus conditions

The chlorophyll content of flag leaves of DH1 population and their parents at the S1, S2, S3, and S4 stages under three phosphorus conditions are shown in **Table 1**, which were decreased gradually during the S1 to S3 and rapidly at the S4 (**Figures 1B, C** **)**. The ANOVA revealed that the difference in chlorophyll content between parents in most stages/environments was statistically (*P <*0.05) and highly significant (*P <*0.01). At the S1 and S2 stages, the male parent Jinmai 84 had more chlorophyll content than the female parent Jinmai 47 under all phosphorus conditions, whereas, at the S3 stage, the chlorophyll content of Jinmai 84 was higher than Jinmai 47 under CK and MP conditions but lower under LP conditions. As well as, at the S4 stage, the chlorophyll content of Jinmai 47 was higher than Jinmai 84 under three different phosphorus conditions, indicating that Jinmai 47 was much more tolerant to low phosphorus stress than Jinmai 84 at the final grain filling stage. During the S1 to S4, the chlorophyll content of parents under CK and MP conditions was higher than that under LP conditions, with the most significant difference occurring at S4. During the S1 to S3, there was no significant difference in chlorophyll content under CK and MP conditions; however, in S4, CK conditions had significantly higher chlorophyll content than MP conditions. The changing regulation of chlorophyll content of the DH population at different stages was similar to that of parents under three phosphorus circumstances (**Figure 1C**).

**Table 1:** The chlorophyll content of flag leaves in parents and DH population derived from Jinmai 47 × Jinmai 84 at four stages under three phosphorus conditions.

Stage	Environments	Parent		DH Population							
		Jinmai 47	Jinmai 84	Mean	SD	Max	Min	Kurtosis	Skewness	CV(%)	*H^2^*
S1	CK1_S1	53.00	58.75*	56.54	2.988	64.88	48.55	-0.232	0.052	5.285	0.8711
	CK2_S1	54.25	57.83*	53.66	2.821	60.80	46.93	-0.474	0.024	5.256	
	AVCK_S1	53.63	58.29*	55.10	2.904	62.84	47.74	-0.353	0.038	5.271	
	MP1_S1	52.85	59.08**	56.20	2.955	62.60	48.75	-0.292	-0.141	5.258	
	MP2_S1	52.20	56.70*	55.31	2.979	64.13	44.70	0.249	-0.142	5.387	
	AVMP_S1	52.53	57.89*	55.75	2.967	63.37	46.73	-0.022	-0.142	5.323	
	LP1_S1	52.65	56.00*	53.85	2.705	61.28	47.03	-0.129	0.334	5.023	
	LP2_S1	51.59	58.73**	53.52	2.696	60.93	47.33	-0.278	0.319	5.038	
	AVLP_S1	52.12	57.36*	53.69	2.701	61.11	47.18	-0.204	0.327	5.030	
	BLUP_S1	53.31	57.34*	54.84	1.831	60.00	50.40	-0.145	0.274	5.034	
S2	CK1_S2	56.05	58.00*	57.60	2.707	64.80	48.78	0.112	-0.117	4.700	0.7090
	CK2_S2	53.90	56.90NS	54.56	2.782	62.23	37.53	6.143	-0.970	5.099	
	AVCK_S2	54.98	57.45NS	56.08	2.745	63.52	43.16	3.128	-0.544	4.900	
	MP1_S2	54.45	58.98**	55.89	2.617	62.80	47.20	0.071	-0.066	4.682	
	MP2_S2	52.65	57.40**	54.79	3.091	61.15	36.15	6.614	-1.431	5.641	
	AVMP_S2	53.55	58.19**	55.34	2.854	61.98	41.68	3.343	-0.749	5.161	
	LP1_S2	52.80	54.55NS	53.44	2.914	60.85	43.03	0.591	-0.033	5.452	
	LP2_S2	54.69	56.50*	52.52	2.972	62.25	37.95	2.594	-0.401	5.658	
	AVLP_S2	53.74	55.53NS	52.98	2.943	61.55	40.49	1.593	-0.217	5.555	
	BLUP_S2	53.39	56.94**	54.80	1.373	58.47	47.83	2.662	-0.414	5.606	
S3	CK1_S3	48.40	55.33**	53.97	3.609	62.20	35.95	4.281	-1.298	6.688	0.6951
	CK2_S3	51.40	52.88NS	52.34	2.940	60.93	43.70	0.373	-0.085	5.617	
	AVCK_S3	49.90	54.10*	53.15	3.275	61.57	39.83	2.327	-0.692	6.152	
	MP1_S3	51.05	54.55*	50.63	5.008	60.38	33.70	0.683	-0.865	9.890	
	MP2_S3	50.85	52.18NS	53.00	3.254	60.43	35.70	9.430	-1.949	6.140	
	AVMP_S3	50.95	53.36*	51.81	4.131	60.41	34.70	5.057	-1.407	8.015	
	LP1_S3	50.05	41.83**	43.22	10.316	57.28	1.63	2.104	-1.463	23.866	
	LP2_S3	47.15	49.88NS	45.94	6.996	59.60	16.65	3.452	-1.562	15.229	
	AVLP_S3	48.60	45.85NS	44.58	8.656	58.44	9.14	2.778	-1.513	19.547	
	BLUP_S3	49.89	51.04NS	49.85	1.887	53.82	42.01	1.793	-0.940	17.388	
S4	CK1_S4	38.55	25.80**	27.97	13.249	54.45	1.98	-0.985	-0.007	47.368	0.6598
	CK2_S4	46.70	25.18**	36.39	12.607	54.58	5.18	-0.793	-0.621	34.644	
	AVCK_S4	42.63	25.49**	32.18	12.928	54.52	3.58	-0.889	-0.314	41.006	
	MP1_S4	27.65	17.53**	19.46	13.092	51.93	1.40	-0.611	0.740	67.266	
	MP2_S4	24.00	14.73**	29.67	15.741	59.03	2.33	-1.431	-0.036	53.054	
	AVMP_S4	25.83	16.13**	24.57	14.417	55.48	1.87	-1.021	0.352	60.160	
	LP1_S4	19.40	7.63**	8.94	7.452	38.70	0.73	1.850	1.405	83.320	
	LP2_S4	15.40	8.35**	16.14	10.751	49.08	0.60	-0.149	0.630	66.628	
	AVLP_S4	17.40	7.99**	12.54	9.102	43.89	0.67	0.851	1.018	74.974	
	BLUP_S4	26.53	16.83**	23.08	5.083	36.05	11.08	-0.330	0.183	70.801	

SD, standard deviation; *H* ^2^, broad-sense heritability; CV, coefficient of variation; BLUP, best linear unbiased prediction; *P <0.05; **P <0.01; NS, not significant.

The heritability of chlorophyll content decreased from 0.8711 to 0.6598 during the S1 to S4, but remained higher than 0.5 (**Table 1**), indicating that genetic factors determined chlorophyll content primarily; as development proceeded, external factors imposed a stronger influence on the phenotypic value. In contrast, the coefficient of variation of chlorophyll content increased from 5.02 to 70.80% (**Table 1**). But, there was no significant difference in the coefficient of variation (CV) of chlorophyll content between the three phosphorus conditions at the S1 and S2 stages. The variance coefficient of chlorophyll content under LP conditions at the S4 was significantly greater than under CK and MP conditions, indicating that low phosphorus stress enhanced the phenotypic variation of chlorophyll content during the middle and later stages of grain filling.

**Figure 2:**
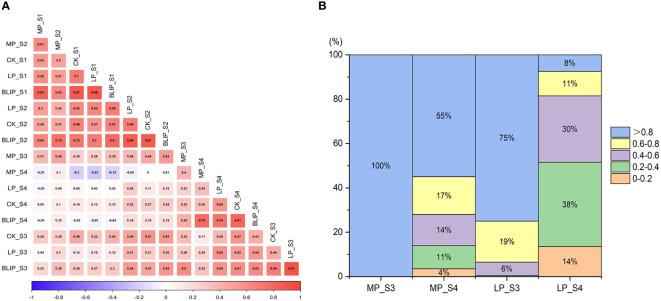
Correlation coefficients of chlorophyll content at four periods under normal, medium, and low phosphorus conditions **(A)**. The LPTC of the S3 and S4 stages under medium and low phosphorus conditions **(B)**.

The correlation coefficient analysis of chlorophyll content at different stages through BLUP value showed a highly significant (*P <*0.01) and positive correlation between each stage within the range of 0.188 and 0.799, except between the S1 and S4 stage. In which, S1 and S2 showed the most highly significant and positive correlation (0.799**), but the S1 and S4 recorded the negative and non-significant relationship (-0.039) (**Table 2**; **Figure 2A**). Under three phosphorus treatments (CK, MP, and LP), the correlation coefficient of chlorophyll content at each stage showed a decreasing trend with the development process (**Tables S3–S5**).

**Table 2:** Correlation coefficients of chlorophyll content between four stages of BLUP data.

	BLUP_S1	BLUP_S2	BLUP_S3
BLUP_S2	0.799**		
BLUP_S3	0.299**	0.554**	
BLUP_S4	-0.039	0.188**	0.623**

**P <0.01.

### Analysis of low phosphorus tolerance coefficient

The higher the value of LPTC, the less phosphorus stress affected the chlorophyll content of flag leaves. At the stage of S3 and S4, with a large variety of chlorophyll content, the LPTC of the DH population was investigated (**Figure 2B**). With development, number of lines with LPTC > 0.8 decreased gradually under MP and LP conditions, and number of lines with LPTC > 0.8 under LP conditions was smaller than MP at the same stage. At S4 stage, 8% of the genotypes exhibited higher LPTC, whereas 14% had lower LPTC under LP conditions, showing variations in phosphorus stress tolerance among DH lines. So, it is necessary to conduct further genetic analysis.

### Construction of genetic linkage map

A genetic linkage map of the DH1 population was constructed by using the Wheat15k SNP panel. The total length of the map was 3,316.06 cM with an average genetic distance of 2.42 cM, including 1,373 SNP markers and across all 21 chromosomes (**Table 3**). The numbers of SNP markers in the A, B, and D genomes were 538, 657, and 178, respectively, and lengths of the linkage were 1,523.80 cM, 1,289.30 cM, and 502.96 cM, with an average distance between markers of 2.83 cM, 1.96 cM, and 2.83 cM, respectively (**Table 3**).

**Table 3:** Summary of linkage group and marker statistics obtained from a 15K SNP panel analysis of the DH1 population (Jinmai 47 × Jinmai 84).

Chromosome	Jinmai 47 × Jinmai 84		
	No. of SNP markers	Length (cM)	Marker density (cM/marker)
1A	94	207.88	2.21
2A	55	178.27	3.24
3A	102	246.17	2.41
4A	52	209.12	4.02
5A	94	327.02	3.48
6A	53	135.82	2.56
7A	88	219.53	2.49
1B	80	185.84	2.32
2B	133	235.12	1.77
3B	120	175.36	1.46
4B	51	136.10	2.67
5B	102	228.71	2.24
6B	100	183.77	1.84
7B	71	144.40	2.03
1D	38	72.05	1.90
2D	33	85.26	2.58
3D	23	99.71	4.34
4D	18	46.93	2.61
5D	26	79.25	3.05
6D	25	68.24	2.73
7D	15	51.52	3.43
A genome	538	1523.80	2.83
B genome	657	1289.30	1.96
D genome	178	502.96	2.83
Total	1373	3316.06	2.42

### QTL mapping for chlorophyll content at four stages under different phosphorus conditions

A total of 157 QTLs were associated with the chlorophyll content of flag leaves under different phosphorus conditions (**Table S6**), and they were distributed across all chromosomes except for 1A, 1B, 3D, 5B, 6D, and 7D. These QTLs explained accounting for 3.07 to 31.66% of PVE with the LOD value ranging from 2.52-18.42. Among them, favorable alleles of 62 QTLs were derived from the female parent Jinmai 47, and favorable alleles of 95 QTLs were derived from the male parent Jinmai 84 (**Table S6**). No QTLs could be expressed consistently in all four stages.

At the S1 stage, a total of 68 QTLs (including BLUP) were detected associated with chlorophyll content, in which 10, 12, and 24 QTLs were identified under CK, MP, and LP conditions, respectively (**Table S6**). The major QTL *Qchl.saw-2B.1* located on chromosome 2B, was detected in four environments (CK1, CK2, MP2, and BLUP), whereas, another major QTL *Qchl.saw-3B.1* positioned on chromosome 3B was detected in three environments (MP2, LP1, and BLUP). As well as, two major QTLs (*Qchl.saw-4D.1* and *Qchl.saw-4D.2*) located on chromosome 4D could be detected under different phosphorus conditions, explaining 3.07-27.31% and 7.36-31.66% of PVE, respectively (**Table 4**).

**Table 4:** Major QTLs for chlorophyll content at four stages under three phosphorus environments.

Stage	Environment	QTL Name	Chr.	LOD	*R^2^* (%)	Add.	Left marker	Right marker	Interval (cM)	Physical interval (Mb)
S1	CK1_S1	*Qchl.saw-2B.1*	2B	3.95	4.13	-0.62	*2B_162775090*	*2B_189706341*	86.1-92.1	162.775/189.706
	CK1_S1	*Qchl.saw-4D.1*	4D	11.14	21.46	-1.74	*4D_101443068*	*4D_98078212*	26.6-28.8	101.443/98.078
	CK1_S1	*Qchl.saw-4D.2*	4D	18.42	31.48	-1.97	*4D_28547729*	*4D_15772687*	37.9-42.4	28.548/15.773
	CK2_S1	*Qchl.saw-2B.1*	2B	4.18	6.69	-0.74	*2B_200710374*	*2B_189706341*	88.9-92.1	200.710/189.706
	CK2_S1	*Qchl.saw-4D.1*	4D	8.93	15.74	-1.14	*4D_101443068*	*4D_98078212*	26.6-29.3	101.443/98.078
	MP1_S1	*Qchl.saw-4D.2*	4D	4.40	7.36	-1.17	*4D_65859359*	*4D_15772687*	34.4-45.9	65.859/15.773
	MP2_S1	*Qchl.saw-2B.1*	2B	3.99	5.74	-0.72	*2B_200710374*	*2B_189706341*	88.6-92.6	200.710/189.706
	MP2_S1	*Qchl.saw-3B.1*	3B	4.37	6.76	0.79	*3B_733329204*	*3B_719457438*	62.3-63.5	733.329/719.457
	MP2_S1	*Qchl.saw-4D.1*	4D	7.51	11.90	-1.08	*4D_101443068*	*4D_98078212*	26.6-28.9	101.443/98.078
	MP2_S1	*Qchl.saw-4D.2*	4D	10.32	17.35	-1.31	*4D_28547729*	*4D_15772687*	38.9-44.7	28.548/15.773
	LP1_S1	*Qchl.saw-3B.1*	3B	2.81	3.95	0.56	*3B_738758982*	*3B_719457438*	60.4-63.5	738.758/719.457
	LP1_S1	*Qchl.saw-4D.1*	4D	7.18	12.04	-0.96	*4D_101443068*	*4D_98078212*	27.2-29.9	101.443/98.078
	LP1_S1	*Qchl.saw-4D.2*	4D	10.16	17.79	-1.17	*4D_28547729*	*4D_15772687*	38.9-45.9	28.548/15.773
	LP2_S1	*Qchl.saw-4D.1*	4D	14.66	27.31	-1.46	*4D_101443068*	*4D_98078212*	26.6-29.0	101.443/98.078
	LP2_S1	*Qchl.saw-4D.2*	4D	17.26	31.66	-1.59	*4D_28547729*	*4D_15772687*	38.9-43.4	28.548/15.773
	BLUP_S1	*Qchl.saw-2B.1*	2B	7.05	11.63	-0.83	*2B_162775090*	*2B_189706341*	88.6-92.6	162.775/189.706
	BLUP_S1	*Qchl.saw-3B.1*	3B	10.99	10.14	0.61	*3B_736664044*	*3B_719457438*	61.0-63.2	736.664/719.457
	BLUP_S1	*Qchl.saw-4D.1*	4D	3.42	3.07	-0.33	*4D_101443068*	*4D_98078212*	26.6-29.0	101.443/98.078
S2	CK1_S2	*Qchl.saw-4D.2*	4D	12.84	22.62	-1.34	*4D_28547729*	*4D_15772687*	38.9-45.9	28.548/15.773
	MP2_S2	*Qchl.saw-4D.1*	4D	6.12	11.13	-1.05	*4D_101443068*	*4D_98078212*	27.2-29.9	101.443/98.078
	MP2_S2	*Qchl.saw-4D.2*	4D	8.99	15.83	-1.25	*4D_48697668*	*4D_15772687*	36.2-43.6	28.5478/15.773
	LP1_S2	*Qchl.saw-4D.1*	4D	8.26	14.54	-1.13	*4D_101443068*	*4D_98078212*	27.2-29.9	101.443/98.078
	LP1_S2	*Qchl.saw-4D.2*	4D	12.18	19.87	-1.33	*4D_28547729*	*4D_15772687*	38.8-42.4	28.548/15.773
	LP2_S2	*Qchl.saw-4D.1*	4D	5.14	9.78	-0.96	*4D_101443068*	*4D_98078212*	27.2-29.3	101.443/98.078
	LP2_S2	*Qchl.saw-4D.2*	4D	7.53	13.96	-1.14	*4D_28547729*	*4D_15772687*	34.4-43.1	28.548/15.773
	BLUP_S2	*Qchl.saw-4D.2*	4D	7.67	4.48	-0.29	*4D_48697668*	*4D_28547729*	36.7-41.1	48.697/28.547
S3	CK1_S3	*Qchl.saw-5A.9*	5A	2.59	5.28	0.85	*5A_645253609*	*5A_613546850*	67.1-83.6	645.254/613.547
	MP2_S3	*Qchl.saw-5A.9*	5A	2.95	6.15	0.82	*5A_645253609*	*5A_613546850*	65.8-83.3	645.254/613.547
	LP2_S3	*Qchl.saw-5A.9*	5A	3.10	5.88	1.71	*5A_645253609*	*5A_613546850*	64.8-82.0	645.254/613.547
	BLUP_S3	*Qchl.saw-5A.9*	5A	6.51	12.6	2.12	*5A_645253609*	*5A_613546850*	66.4-83.7	645.254/613.547
S4	CK1_S4	*Qchl.saw-6A.4*	6A	2.58	4.83	2.98	*6A_69374520*	*6A_61743025*	49.3-52.2	69.375/61.743
	MP2_S4	*Qchl.saw-6A.4*	6A	3.22	5.89	3.89	*6A_84693688*	*6A_69374520*	47.1-49.3	84.693/69.375
	BLUP_S4	*Qchl.saw-6A.4*	6A	4.33	11.27	4.71	*6A_69374520*	*6A_64071530*	49.3-51.7	69.375/64.071

At the stage of S2, 43 QTLs (including BLUP) with chlorophyll content were detected, of which 14, 6, and 13 QTLs were detected under CK, MP, and LP conditions, respectively (**Table S6**). None of the QTL was detected that could be expressed under the three phosphorus conditions. Major QTL *Qchl.saw-4D.1* and *Qchl.saw-4D.2* could be detected under MP and LP conditions, exhibiting 14.54% and 19.87% of PVE, respectively (**Table 4**).

At the S3, in total 19 QTLs were detected, and *Qchl.saw-5A.9* was detected as major due to identified in four environments, accounting for 5.30 to 12.60% of the PVE. At the S4, a total of 27 QTLs of chlorophyll content were detected, of which *Qchl.saw-6A*.4 was detected in three environments, with up to 11.27% of PVE (**Tables 4**; **S6**).

### Additive effects of the six major QTLs on chlorophyll content

**Figure 3:**
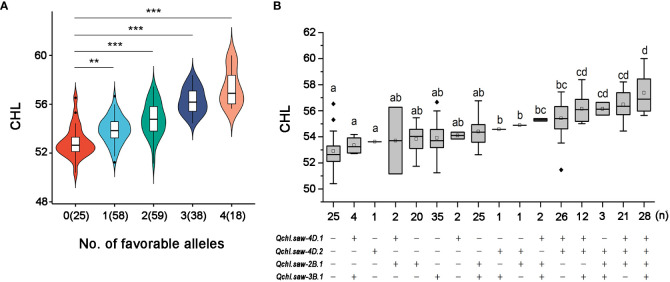
Relationship of numbers of favorable alleles and chlorophyll content in the DH1 population **(A)**. Linear regressions between phenotypes and additive effects of the major QTLs for chlorophyll content in the DH1 population **(B)**. ** and *** represent significance at *P <*0.01 and *P <*0.001, respectively. The letter above the bars indicated comparisons result at the significant level of 0.05 and respectively.

At S1, four major QTLs (*Qchl.saw-2B.1*, *Qchl.saw-3B.1*, *Qchl.saw-4D.1*, and *Qchl.saw-4D.2*) were detected (**Table 4**), and their additive effects for chlorophyll content (BLUP) were determined by using their linkage markers, which revealed that the more favorable alleles for polymerization, and higher the chlorophyll content (**Figure 3A**). The chlorophyll content of DH lines with 4, 3, 2, and 1 favorable allele increased by 8.45%, 6.41%, 3.52%, and 1.87% compared to those without four favorable alleles (*P <*0.05) (**Table S7**). The chlorophyll content of lines with only *Qchl.saw-4D.1* allele was higher than that of other lines with only one favorable allele, implying that *Qchl.saw-4D.1* had the strongest genetic effect on chlorophyll content (**Table S7**; **Figure 3B**). In addition, *Qchl.saw-4D.1* (S2) and *Qchl.saw-4D.2* (S2), *Qchl.saw-5A.9* (S3) and *Qchl.saw-6A.4* (S4) had strongly influenced chlorophyll content in more than three environments at different stages (**Figure S2**).

### Validation of the major and stable QTLs *Qchl.saw-4D.1* and *Qchl.saw-4D.2*

**Figure 4:**
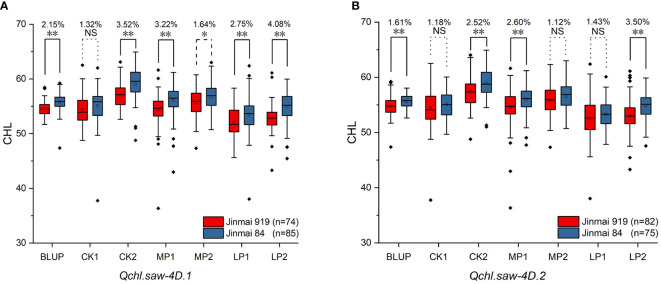
Validation of *Qchl.saw-4D.1* **(A)**, and *Qchl.saw-4D.2* **(B)** in DH2 (Jinmai 919 × Jinmai 84) population under different environments. ** Significant at *P* <0.01; * Significant at *P* <0.05; NS, not significant.

Six major QTLs *Qchl.saw-2B.1*, *Qchl.saw-3B.1*, *Qchl.saw-4D.1*, *Qchl.saw-4D.2*, *Qchl.saw-5A.9* and *Qchl.saw-6A.4* were detected under multiple environments (**Table 4**). The favorable alleles of *Qchl.saw-3B.1*, *Qchl.saw-5A.9*, and *Qchl.saw-6A.4* were derived from the female parent Jinmai 47, whereas the favorable alleles of *Qchl.saw-2B.1*, *Qchl.saw-4D.1*, and *Qchl.saw-4D.2* were derived from the male parent Jinmai 84. To further validate the *Qchl.saw-2B.1*, *Qchl.saw-4D.1*, and *Qchl.saw-4D.2* in DH2 population, peak markers for each QTL were employed to evaluate their effects on chlorophyll content. The peak marker for *Qchl.saw-2B.1* was not polymorphic between the Jinmai 919 and Jinmai 84, and thus the effects could not be evaluated. The remaining two QTLs (*Qchl.saw-4D.1* and *Qchl.saw-4D.2*) were validated in the DH2 population (**Figure 4**). The effect of *Qchl.saw-4D.1* for chlorophyll content was significant (*P <*0.05) under MP2 and highly significant (*P <*0.01) under CK2, MP1, LP1, LP2, and BLUP conditions. The effect of *Qchl.saw-4D.2* was highly significant (*P <*0.01) under CK2, MP1, LP2, and BLUP conditions.

### Predictive analysis of candidate genes of major QTLs *Qchl.saw-2B.1, Qchl.saw-3B.1, Qchl.saw-4D.1, Qchl.saw-4D.2, Qchl.saw-5A.9* and *Qchl.saw-6A.4*

**Figure 5:**
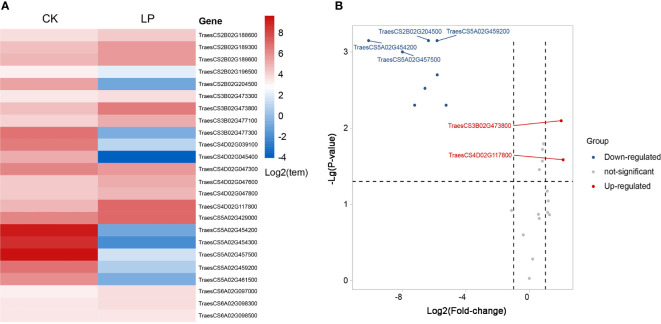
Differential expression heat map of chlorophyll genes screened under different phosphorus stress conditions **(A)**. Volcano plot of differentially expressed genes under phosphorus stress conditions **(B)**. Red indicates up-regulated genes, and blue indicates down-regulated genes.

The genomic functional annotation was performed between marker intervals of six major QTLs *Qchl.saw-2B.1* (162.77-189.70 Mb), *Qchl.saw-3B.1* (719.45-736.66 Mb), *Qchl.saw-4D.1* (98.07-101.44 Mb), *Qchl.saw-4D.2* (15.77-28.54 Mb), *Qchl.saw-5A.9* (613.54-645.25 Mb) and *Qchl.saw-6A.4* (61.74-69.37 Mb), and a total of 235 genes were identified to be associated with phosphorus and chlorophyll function enriched (**Table S8**; **Figure S3**). The expression patterns of these genes in leaves were analyzed by the expVIP platform, and the number of highly expressed genes in the above QTLs interval was 5, 4, 1, 5, 6, and 3, respectively, including 11 genes related to chlorophyll and photosynthesis and 13 genes associated with phosphorus (**Table 5**; **Figure 5A**).

**Table 5:** Function genes related to chlorophyll and phosphorus, which highly expressed in leaves.

Chr.	Start (bp)	Stop (bp)	Ori.	Name	Function
chr2B	164088025	164091566	–	*TraesCS2B02G188600*	nucleic acid binding
chr2B	164936500	164940359	+	*TraesCS2B02G189300*	chloroplast
chr2B	165115340	165119793	–	*TraesCS2B02G189600*	ATP binding, protein phosphorylation
chr2B	174591804	174593791	–	*TraesCS2B02G196500*	biosynthetic process
chr2B	184326362	184332008	+	*TraesCS2B02G204500*	oxidoreductase activity, acting on the CH-OH group of donors, NAD or NADP as acceptor
chr3B	722475646	722480010	–	*TraesCS3B02G473300*	ATP binding
chr3B	723031626	723035032	–	*TraesCS3B02G473800*	pentose-phosphate shunt, chloroplast stroma, carbohydrate metabolic process
chr3B	725577635	725581339	–	*TraesCS3B02G477100*	pyridoxal phosphate binding, biosynthetic process
chr3B	725707258	725708766	+	*TraesCS3B02G477300*	chloroplast thylakoid membrane, chloroplast
chr4D	98441989	98444445	+	*TraesCS4D02G117800*	lipid metabolic process
chr4D	16797472	16799127	+	*TraesCS4D02G039100*	nucleic acid binding
chr4D	20897901	20899133	+	*TraesCS4D02G045400*	photosynthesis, photosystem II oxygen-evolving complex
chr4D	22938811	22945799	+	*TraesCS4D02G047300*	carbohydrate metabolic process
chr4D	23412775	23419191	+	*TraesCS4D02G047600*	GTP binding
chr4D	23420023	23426081	–	*TraesCS4D02G047800*	chloroplast, ATP synthesis coupled electron transport
chr5A	613539449	613543349	+	*TraesCS5A02G429000*	ATP binding
chr5A	635407573	635408909	–	*TraesCS5A02G454200*	chloroplast thylakoid membrane, chlorophyll binding
chr5A	635410443	635411662	–	*TraesCS5A02G454300*	chloroplast thylakoid membrane, chlorophyll binding
chr5A	637167477	637168439	+	*TraesCS5A02G457500*	photosynthesis
chr5A	639306149	639307856	–	*TraesCS5A02G459200*	chloroplast stroma, photosynthesis
chr5A	641732244	641734013	+	*TraesCS5A02G461500*	photosynthesis
chr6A	64255216	64262082	+	*TraesCS6A02G097000*	an integral component of membrane
chr6A	65434223	65437470	+	*TraesCS6A02G098300*	lipid metabolic process
chr6A	65681324	65684847	–	*TraesCS6A02G098500*	an integral component of membrane

Genes related to chlorophyll and photosynthesis regulate the structural composition of chloroplast, chloroplast stroma, thylakoid membrane, and a series of processes of photosynthesis, as well as the formation of photosystem II oxygen evolution complex to affect photosynthesis. Phosphorus-related genes with high levels of expression respond to phosphorus stress by regulating ATP, GTP, and NAD+ binding. The ATP synthesis is coupled with proton transport and protein phosphorylation, carbohydrate metabolism, biosynthesis, pyridoxal phosphate binding, lipid metabolism, and nucleic acid binding. The differential expression of 24 genes under normal and low phosphorus conditions, which was established by the expVIP platform in order to screen the genes controlling chlorophyll content under phosphorus stress more accurately. Two genes were up-regulated, and four were down-regulated in response to low phosphorus conditions (**Figure 5B**). The gene *TraesCS3B02G473800* belongs to the QTL *Qchl.saw-3B.1* and which is homologous to the *TRA2* gene of *Arabidopsis thaliana* and regulates pentose-phosphate shunt, chloroplast stroma, and carbohydrate metabolic process by up-regulating expression, it has a similar function in *Arabidopsis chloroplast* (Peltier et al., 2006), whereas gene *TraesCS4D02G117800* in the major QTL *Qchl.saw-4D.1* interval responds to low phosphorus stress by up-regulating phospholipid metabolism. However, its homologous gene *Os11g0112800* in rice shows high expression in the process of controlling flower buds and rooting after anthesis (Kikuchi et al., 2003). The down-regulated expression of gene *TraesCS2B02G204500* decreased the oxidoreductase activity and thus decreased the transformation of NAD or NADP were deceived in *Qchl.saw-2B.1*. Previous studies have found that the homologous gene *Os07g0601100* in rice was regulated the process of anthocyanin reduction, and has high expressed in rice leaves after anthesis, which echoes the chlorophyll-related QTL were located after anthesis in wheat (Kikuchi et al., 2003). While *Qchl.saw-5A.9* interval’s genes *TraesCS5A02G454200*, *TraesCS5A02G457500*, and *TraesCS5A02G459200* decreased chlorophyll content and photosynthesis by down-regulating the synthesis of chloroplast matrix and chloroplast thylakoid membrane in wheat, in which the gene *TraesCS5A02G459200* is homologous of *RbcX2* regulating the synthesis of chloroplast matrix in *Arabidopsis thaliana* and *Os03g0807900* regulating chloroplast synthesis in rice. We are gratified that these homologous genes show the same function in different crops (Kikuchi et al., 2003; Kolesinski et al., 2011).

## Discussion

### Effect of low phosphorus stress on the dynamic change of chlorophyll content

There is no statistically significant change in chlorophyll content between DH populations under three phosphorus conditions during the S1 and S2 stages (**Table 1**). Because phosphorus is a reusable element, and up to 75% of the phosphorus in leaves is in the form of orthophosphate, of which 85% is present in vacuoles, which act as a buffer for phosphorus storage (Richardson et al., 2011; Van de Wiel et al., 2016). When phosphorus deficiency occurs at the early filling stage, phosphorus in the vacuole is allocated preferentially to mesophyll cells to ensure the synthesis of photosynthetic proteins (Conn and Gilliham, 2010; Hayes et al., 2018), the stability of chloroplasts and chlorophyll, and thus the maintenance of high photosynthetic efficiency (Veneklaas et al., 2012). At the middle and later stages of grain filling, the photosynthetic organ will transport phosphorus to the grain more rapidly. When phosphorus supply is adequate, 65% of phosphorus in photosynthetic organs will be transported to grains, whereas under conditions of phosphorus deficiency, 85% of phosphorus in photosynthetic organs will be transferred to grains (El Mazlouzi et al., 2020). The greater the degree of phosphorus deficiency, the more serious the senescence of photosynthetic organs. Therefore, the chlorophyll content of flag leaves of the DH population at the S3 and the S4 stages under LP conditions was significantly lower than under CK conditions. At the last stage of grain filling, phosphorus deficiency not only reduces the chlorophyll content and photosynthetic area of wheat (Khourchi et al., 2022; Nguyen et al., 2022) but also influences the photosynthetic efficiency (Nguyen et al., 2022), resulting in a decrease in the number of ATP (Lin et al., 2009), decreasing the synthesis of RuBP, limiting the fixation of CO_2_ (Bown et al., 2009), and seriously affecting yield formation (McDonald et al., 2015).

### QTL expression characteristics of chlorophyll content at different stages under different phosphorus conditions

QTL analysis was performed on the chlorophyll content of flag leaves at different filling stages under normal, medium, and low phosphorus conditions using the DH population derived from Jinmai 47 and Jinmai 84. A total of 68, 43, 19, and 27 QTLs for chlorophyll content were detected at the S1, S2, S3, and S4 stages, respectively (**Table S6**), indicating that the genes controlling chlorophyll content were expressed most at the early filling stage, and gradually decreased as development progressed, which was consistent with the phenotypic change tendency of chlorophyll content.

The majority of the chlorophyll content QTLs detected at various stages were different, and no QTLs could be expressed throughout the filling stage. The QTL viz. *Qchl.saw-2B.1* (S1 and S2), *Qchl.saw-3B.1* (S1 and S3), *Qchl.saw-4A.5* (S2 and S3), *Qchl.saw-4D.1* (S1 and S2), and *Qchl.saw-4D.2* (S1 and S2) could be detected at two different stages (**Table S6**). Yang et al. (2016b) reported a similar phenomenon in the QTL analysis of chlorophyll content at different filling stages, indicating that the expression of genes controlling chlorophyll content displayed certain both temporal and spatial characteristics, i.e., the majority of genes were only expressed temporarily in one or more developmental stages (Zhang et al., 2011). In other words, detecting the QTL of chlorophyll content in flag leaves at multiple filling stages helps to identify more major QTLs.

This study identified 36, 30, and 48 chlorophyll-containing QTLs under CK, MP, and LP conditions, respectively (**Table S6**). The number of QTLs detected under LP conditions was much larger than under CK conditions, implying that low phosphorus stresses may induce the expression of genes silenced under normal phosphorus conditions. Similarly, previous research has been reported (Su et al., 2009; Vijayalakshmi et al., 2010; Yang et al., 2016b). The majority of chlorophyll content QTLs detected under CK, MP, and LP conditions are different, indicating that the chlorophyll regulation mechanism under low phosphorus conditions may differ from that under normal phosphorus conditions, and similar phenomena have been observed in previous research (Yang et al., 2018; Yuan et al., 2020; Yang et al., 2021). Under multiple phosphorus conditions, six major QTLs *Qchl.saw-2B.1*, *Qchl.saw-3B.1*, *Qchl.saw-4D.1*, *Qchl.saw-4D.2*, *Qchl.saw-5A.9* and *Qchl.saw-6A.4* were detected (**Table 4**). Hassan et al. (2018) hypothesized that the major QTLs identified under stress conditions may contain stress-resistant functional genes that enhance yield potential. Wang et al. (2019b) also suggested that mapping QTL in phosphorus stress environments and identifying the major QTLs that are less influenced by environmental conditions is very important for low phosphorus tolerance breeding. Under the LP conditions, both major QTLs *Qchl.saw-4D.1* and *Qchl.saw-4D.2* could be detected, and their contribution to phenotype was more than 20%. It has the potential to be utilized in molecular marker-assisted breeding of wheat for low phosphorus tolerance.

### Comparison with the results of previous studies

Chlorophyll synthesis and degradation is a complex biological process, and external and internal factors easily influence the regulation of chlorophyll gene expression. Before being applied to MAS breeding, six major QTLs detected under different phosphorus conditions should be validated in populations with different genetic backgrounds to confirm that they are not affected by the genetic background and are closely connected to the target traits (Ahmed et al., 2021). A total of 6, 17, 1, 14, and 9 QTLs for chlorophyll content were located on chromosomes 2B, 3B, 4D, 5A, and 6A, respectively, in previous studies (**Table 6**). The *Qchl.saw-2B.1* was located between 162.77 Mb to 189.70 Mb on chromosome 2B. In the same interval, a chlorophyll content QTL *qChlN-2B* with an 8.3% phenotypic contribution rate was identified (Li et al., 2010), and a major QTL *IWB29808* for 1000-grain weight was identified (Zhang et al., 2018). The *Qchl.saw-3B.1* was identified on chromosome 3B between 719.45 Mb and 739.66 Mb, and a flag leaf chlorophyll content QTL, *Qspad.acs-3B.2*, was detected in the same location (Yang et al., 2016b). The *Qchl.saw-5A.9* was identified between 613.54 Mb and 645.25 Mb on chromosome 5A. In the same interval, a major QTL for chlorophyll content in flag leaves, *Qchl.saw-5A.3* (Yang et al., 2022), and three QTL for 1000-grain weight, *QTKW.ndsu.5A.2* (Kumar et al., 2016), *TKW1-IWB686* (Sukumaran et al., 2015), and *TKW-IWB789* (Zhang et al., 2018) were detected, and a nitrogen efficiency gene *TaNAC2-5A* was cloned (He et al., 2015). The *Qchl.saw-6A.4* was identified in the interval of 61.74 Mb to 69.37 Mb on chromosome 6A. However, no chlorophyll content QTLs were detected in this location previously, indicating that *Qchl.saw-6A.4* is a novel major QTL of chlorophyll content, and a major QTL *QTkw-6A.1* for 1000-grain weight was detected by Cui et al. (2014) in the same interval.

**Table 6:** Chlorophyll QTLs on chromosomes 2B, 3B, 4D, 5A, and 6A from previous studies.

Chr.	Left marker	Right marker	Physical interval (Mb)	Reference
2B	*WMC441*	*WMC344*	165.58-598.06	Li et al., 2010
2B	*Xwmc223*	*Xbarc101*	240.22-621.47	Yang et al., 2016b
2B	*Xcfa2278*	*Xgwm55*	406.54	Yang et al., 2016b
2B	*Xpsp3034*	*Xgwm630*	442.79	Li et al., 2010
2B	*Xgwm55*	*Xbarc128*	523.79	Yang et al., 2016b
2B	*Xgwm388*	*Xmag3319*	557.37	Yang et al., 2016b
3B	*wsnp_Ra_c41135_48426638*	*wsnp_BE497169B_Ta_2_1*	3.41-16.04	Shirdelmoghanloo et al., 2016
3B	*Xbarc087*	*Xaag/ctc-1*	14.39	Tahmasebi et al., 2017
3B	*Xwmc808*	*Xbarc102*	17.57-42.71	Yang et al., 2016b
3B	*Xgwm533*	*Xgwm1037*	35.32-77.72	Kumar et al., 2010
3B	*Xwmc689*	*Xwmc78*	43.68-201.87	Puttamadanayaka et al., 2020
3B	*BS00010818_51*	*Excalibur_c8284_580*	52.83-54.76	Yang et al., 2022
3B	*Xgwm264*	*Xgwm566*	68.91-77.72	Awlachew et al., 2016
3B	*Xbarc68*	*Xbarc101*	76.13-621.47	Kumar et al., 2012
3B	*Xgwm566*	*Xwmc540*	77.72-132.94	Yang et al., 2016b
3B	*Xgwm566*	*Xgwm72*	77.72-216.62	Li et al., 2010
3B	*Xgwm566*	*Xgwm285*	77.72-415.92	Yang et al., 2016a
3B	*IWB10755*	*-*	238.82	Maulana et al., 2020
3B	*Xbarc358*	*-*	622.38	Ilyas et al., 2020
3B	*Xmag3356*	*Xwmc291*	700.81-808.66	Yang et al., 2016b
3B	*Xwmc326*	*-*	778.70	Barakat et al., 2015
3B	*wsnp_Ex_c16569_25082817*	*Tdurum_contig31097_254*	811.45-817.82	Yang et al., 2022
3B	*Xgwm340*	*wPt8352*	826.23	Ballesteros et al., 2015
4D	*Xgwm192*	*WMC331*	453.33	Li et al., 2010
5A	*wsnp_Ku_c7890_13514597*	*wsnp_Ex_c9842_16228523*	15.85-19.23	Yang et al., 2022
5A	*Xgwm154*	*Xgwm156*	21.00-450.16	Yang et al., 2016a
5A	*Xgwm443*	*P2470-280*	22.71-105.43	Li et al., 2010
5A	*wpt8226*	*tpt9702*	85.15	Ghaedrahmati et al., 2014
5A	*tpt9702*	*gwm415*	85.15	Ghaedrahmati et al., 2014
5A	*Xgwm415*	*Xgwm304*	105.43	Yang et al., 2007
5A	*AX-94414339*	*AX-94730618*	556.01-561.11	Puttamadanayaka et al., 2020
5A	*wsnp_Ra_c3414_6378271*	*Kukri_c61046_510*	569.55-582.39	Yang et al., 2022
5A	*RAC875_rep_c109716_67*	*IACX448*	586.60-588.38	Yang et al., 2022
5A	*GENE-2735_151*	*RAC875_c79540_228*	586.60-615.31	Yang et al., 2022
5A	*Xwmc410*	*Xgwm595*	678.29-680.07	Yang et al., 2016a
5A	*Xgwm415*	*wPt9452*	692.78	Ballesteros et al., 2015
5A	*WMC74*	*Xgwm291*	702.50	Yang et al., 2007
5A	*Xbarc122*	*-*	766.16	Barakat et al., 2015
6A	*Xgwm334*	*-*	9.25	Barakat et al., 2015
6A	*aca/caa-5*	*wPt-7599*	32.67	Hassan et al., 2018
6A	*Xgdm36*	*-*	94.31	Li et al., 2012
6A	*Xb*arc171	*Xgwm427*	354.59	Yang et al., 2016b
6A	*Xgwm570*	*Xpsp3071*	447.12-579.13	Yang et al., 2016a
6A	*Xwmc201*	*Xwmc684*	461.84	Yang et al., 2016b
6A	*Xbarc113*	*Xwmc621*	495.11	Yang et al., 2016b
6A	*Xgwm169*	*Xwmc580*	595.38	Yang et al., 2016b
6A	*Xgwm427*	*Xgwm169*	595.38	Ilyas et al., 2014

Spanning markers were used to locate positions on the physical map if certain markers failed to be located on the physical map. The physical locations of some markers were not available, leaving the physical location as a single marker.

The donor of the wheat D genome is the species of *Aegilops tauschii*. Previous research has revealed that the D genome of wheat plays a crucial role in stress tolerance (Mirzaghaderi and Mason, 2019; Hussain et al., 2022). In this study, the two major chlorophyll QTLs with the highest phenotypic contribution rate were identified on chromosome 4D, and there was no previously reported chlorophyll content QTLs in same interval, suggesting that *Qchl.saw-4D.1* and *Qchl.saw-4D.2* may be novel major QTL. Furthermore, *Qchl.saw-4D.1* and *Qchl.saw-2D.2* have been validated by the DH2 population with different genetic backgrounds, so they may be less affected by genetic background. Interestingly, *Qchl.saw-4D.2* was identified in the 15.77 Mb-28.54 Mb interval, which also contained two major QTLs, viz. *QTKW-4D-AN* (Mohler et al., 2016) and *QTgw-4D* (Liu et al., 2014) were associated with 1000-grain weight. Dixon et al. (2018) identified the gene *TB-D1*, which regulates the structure of wheat inflorescence at the position 18.46 Mb on chromosome 4D. Peng et al. (1999) reported that the green revolution dwarf gene *Rht-D1* was positioned at the location of 18.78 Mb on the linkage group 4D. The QTLs/genes of yield-related traits such as chlorophyll content and 1000-grain weight have been co-located in the same interval as in earlier studies using various genetic backgrounds, confirming that the major QTLs in this study have significant potential for application in marker-assisted selection (MAS) program of wheat. In summary, the major QTLs *Qchl.saw-4D.1* and *Qchl.saw-4D.2* as well as the developed KASP markers identified in this study could be utilized in developing low-phosphorus tolerant varieties of wheat.

## Data availability statement

The original contributions presented in the study are included in the article/**Supplementary Material**. Further inquiries can be directed to the corresponding authors.

## Author contributions

JL, LQ, and BY designed the experiment and wrote the manuscript. BY, NC and YD carried out the experiments. JZ analyzed the data. YW, XZ, JJZ, and HW did the field experiments. All authors contributed to the manuscript and approved the final manuscript to publish.

## Funding

The research project is supported by the Basic Research Program of Shanxi Province (202103021223156 and 20210302124505), the Central Leading Local Technology Development Fund Project of Shanxi Province (YDZJSX2022A033), and the Shanxi Scholarship Council of China (2020-159).

## Conflict of interest

The authors declare that the research was conducted in the absence of any commercial or financial relationships that could be construed as a potential conflict of interest.

## Publisher’s note

All claims expressed in this article are solely those of the authors and do not necessarily represent those of their affiliated organizations, or those of the publisher, the editors and the reviewers. Any product that may be evaluated in this article, or claim that may be made by its manufacturer, is not guaranteed or endorsed by the publisher.
