# Supplementary material for: Identification and validation of quantitative trait loci for chlorophyll content of flag leaf in wheat under different phosphorus treatments
Source: Front Plant Sci. 2022 Nov 17;13:1019012. doi: 10.3389/fpls.2022.1019012 (PMC9714299; doi:10.3389/fpls.2022.1019012)
Supplement: Supplementary file 1 [file DataSheet_1.doc]

## Supplementary Material

**Supplementary Figures and Tables**

**Supplementary Figure 1.** Variation of temperature and precipitation after flowering.

**Supplementary Figure 2.** Student’s *t*-test against the two groups of lines from the DH1 population carrying the allele from either Jinmai 47 or Jinmai 84 of the major QTLs for chlorophyll content. * and ** represent significance at *P* < 0.05 and *P* < 0.01, respectively; NS, not significant. Differences between the two groups of lines were labeled above the significance levels.

**Supplementary Figure 3.** Function genes related to chlorophyll and phosphorus of the six major QTLs.

**Supplementary Table S1.** Nutrient content of the soil under different phosphorus conditions.

**Supplementary Table S2.** Primer sequences of the KASP markers of *Qchl.saw-4D.1* and *Qchl.saw-4D.2.*

**Supplementary Table S3.** Correlation coefficients for chlorophyll content among four stages under normal phosphorus (CK) conditions.

**Supplementary Table S4.** Correlation coefficients for chlorophyll content among four stages under medium phosphorus (MP) conditions.

**Supplementary Table S5.** Correlation coefficients for chlorophyll content among four stages under low phosphorus (LP) conditions.

**Supplementary Table S6.** Quantitative trait locis (QTLs) for the chlorophyll content were detected in the DH1 population at four stages under three phosphorus conditions.

**Supplementary Table S7.** Additive effects of the four major QTLs at the S1 stage.

**Supplementary Table S8.** Functional annotation and enrichment of genes of the six major QTLs**.**

**Supplementary Figures**


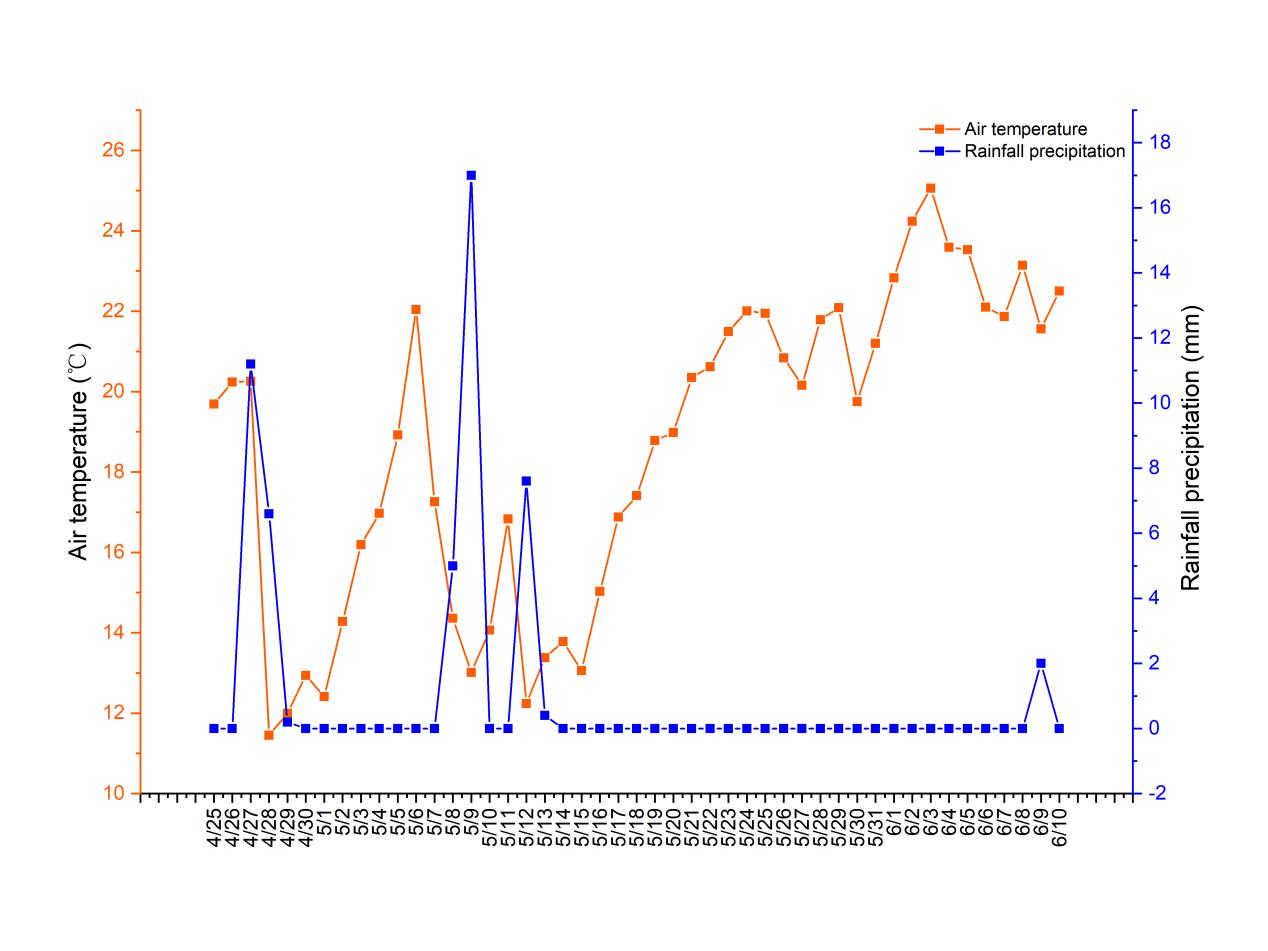


**Figure. S1** Variation of temperature and precipitation after flowering.


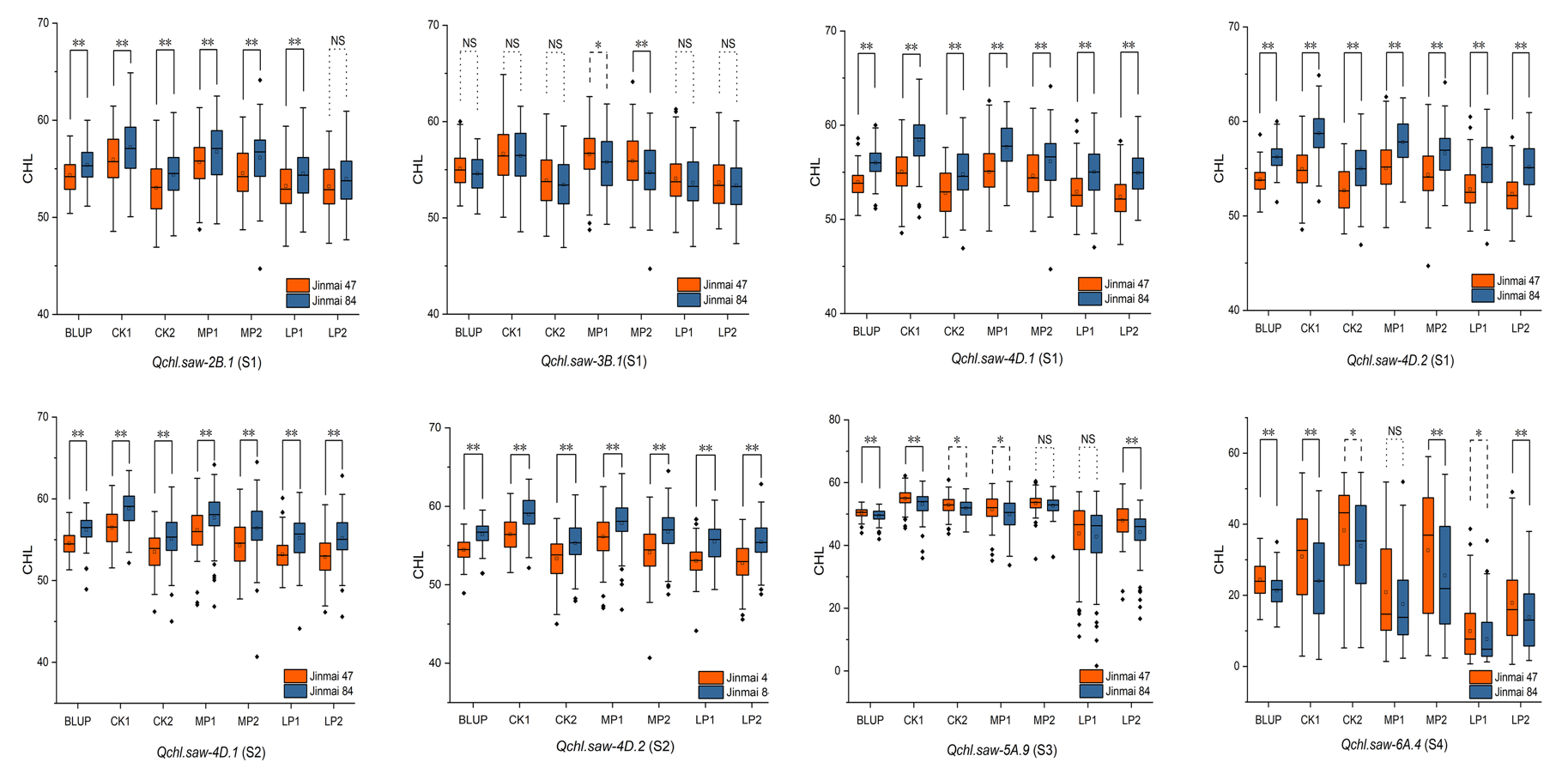


**Figure. S2** Student’s *t*-test against the two groups of lines from the DH1 population carrying the allele from either Jinmai 47 or Jinmai 84 of the major QTLs for chlorophyll content. * and ** represent significance at *P* < 0.05 and *P* < 0.01, respectively; NS, not significant. Differences between the two groups of lines were labeled above the significance levels.


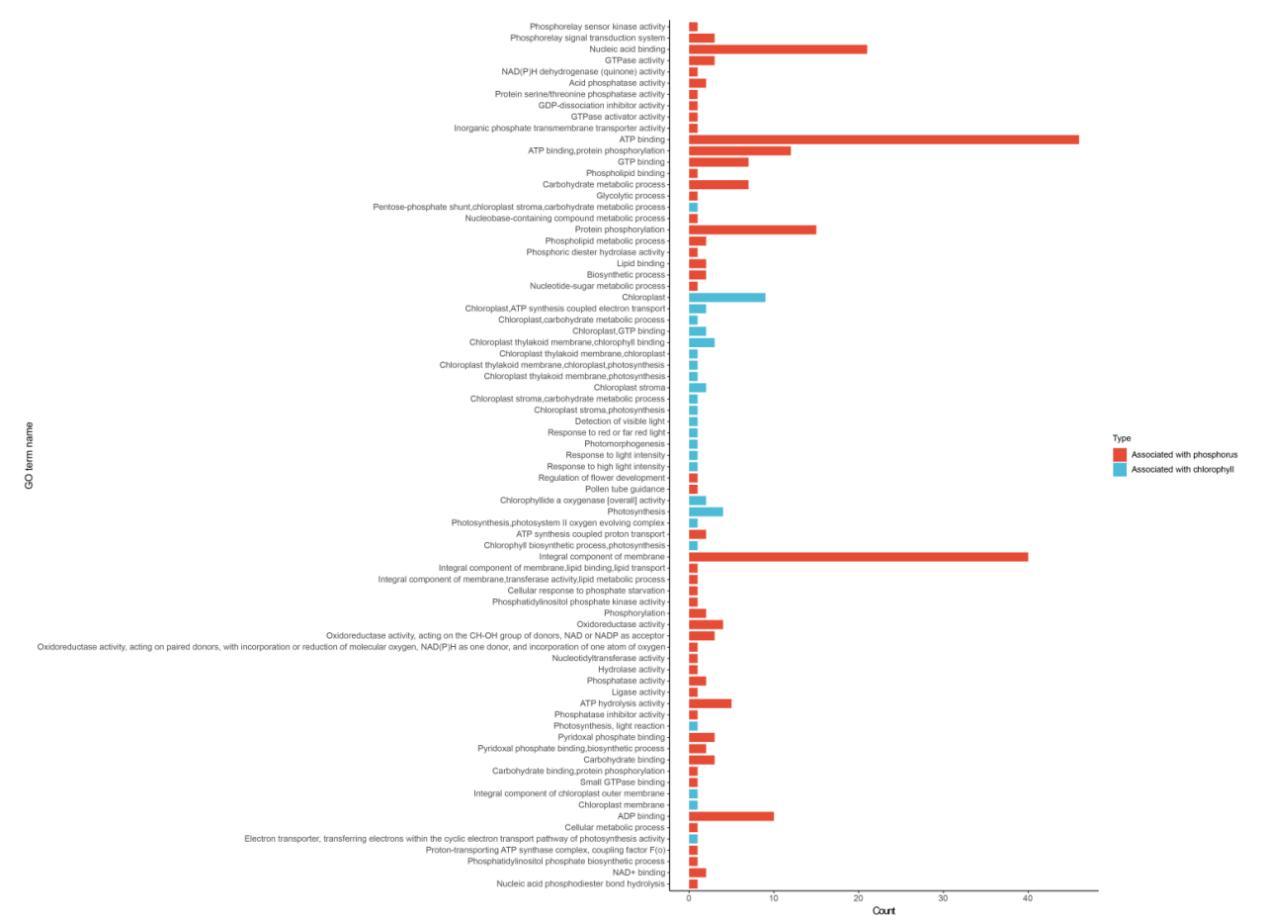


**Figure. S3** Function genes related to chlorophyll and phosphorus of the six major QTLs.

**Supplementary Tables**

**Table S1.** Nutrient content of the soil under different phosphorus conditions.

| Treatment | N (%) | P (mg·kg^-1^) | K (mg·kg^-1^) | SOM (g·kg^-1^) | pH | EC (μs·cm^-1^) |
| --- | --- | --- | --- | --- | --- | --- |
| CK  MP  LP | 0.1  0.1  0.1 | 10.9  6.1  3.5 | 190  196  192 | 23.8  23.8  23.4 | 8.21  8.25  8.17 | 125.3  120.2  130.8 |

**Table S2. Primer sequences of the KASP markers of *Qchl.saw-4D.1* and *Qchl.saw-4D.2.***

| QTL | Marker | Sequence(5'-3') |
| --- | --- | --- |
| *Qchl.saw-4D.1* | FAM | GGTTGTGATATTGTGCTTGATGT |
|  | HEX | GGTTGTGATATTGTGCTTGATGC |
|  | Common | CCACCTTCTACGGCATGGG |
| *Qchl.saw-4D.2* | FAM | GGGCAAGGAGGAGGGAGA |
|  | HEX | GGGCAAGGAGGAGGGAGG |
|  | Common | CCATGACGGACTGATCGCTA |

**Table S3. Correlation coefficients for chlorophyll content among four stages under normal phosphorus (CK) conditions.**

|  | CK_S1 | CK_S2 | CK_S3 |
| --- | --- | --- | --- |
| CK_S2 | 0.661** |  |  |
| CK_S3 | 0.462** | 0.569** |  |
| CK_S4 | 0.137 | 0.266** | 0.567** |

** represent significance at *P* < 0.01.

**Table S4. Correlation coefficients for chlorophyll content among four stages under medium phosphorus (MP) conditions.**

|  | MP_S1 | MP_S2 | MP_S3 |
| --- | --- | --- | --- |
| MP_S2 | 0.607** |  |  |
| MP_S3 | 0.373** | 0.480** |  |
| MP_S4 | -0.066 | 0.104 | 0.399** |

** represent significance at *P* < 0.01.

**Table S5. Correlation coefficients for chlorophyll content among four stages under low phosphorus (LP) conditions.**

|  | LP_S1 | LP_S2 | LP_S3 |
| --- | --- | --- | --- |
| LP_S2 | 0.644** |  |  |
| LP_S3 | 0.149* | 0.411** |  |
| LP_S4 | 0.028 | 0.264** | 0.576** |

* and ** represent significance at *P* < 0.05 and *P* < 0.01, respectively.

**Table S6.** Quantitative trait loci (QTLs) for the chlorophyll content were detected in the DH1 population at four stages under three phosphorus conditions.

| Stage | Environment | QTL Name | Chr. | LOD | *R^2^*(%) | Add. | Left Marker | Right Marker | Interval (cM) | Physical Interval (Mp) |
| --- | --- | --- | --- | --- | --- | --- | --- | --- | --- | --- |
| S1 | CK1_S1 | *Qchl.saw-2B.1* | 2B | 4.00 | 4.13 | -0.62 | 2B_162775090 | 2B_189706341 | 86.1-92.1 | 162.775/189.706 |
|  | CK1_S1 | *Qchl.saw-3A.1* | 3A | 4.55 | 4.88 | -0.66 | 3A_658657616 | 3A_650566332 | 143.5-146.7 | 658.658/650.566 |
|  | CK1_S1 | *Qchl.saw-3A.2* | 3A | 5.70 | 6.03 | -0.74 | 3A_686724449 | 3A_688580956 | 154.7-158.5 | 686.724/688.581 |
|  | CK1_S1 | *Qchl.saw-4B.1* | 4B | 3.88 | 4.02 | -0.61 | 4B_654436643 | 4B_658157524 | 115.5-124.8 | 654.437/658.158 |
|  | CK1_S1 | *Qchl.saw-4D.1* | 4D | 11.14 | 21.46 | -1.74 | 4D_101443068 | 4D_98078212 | 26.6-28.8 | 101.443/98.078 |
|  | CK1_S1 | *Qchl.saw-4D.2* | 4D | 18.42 | 31.48 | -1.97 | 4D_28547729 | 4D_15772687 | 37.9-42.4 | 28.548/15.773 |
|  | CK2_S1 | *Qchl.saw-2B.2* | 2B | 3.95 | 6.34 | -0.72 | 2B_403581918 | 2B_383141007 | 79.4-81.3 | 403.582/383.141 |
|  | CK2_S1 | *Qchl.saw-2B.1* | 2B | 4.18 | 6.69 | -0.74 | 2B_200710374 | 2B_189706341 | 88.9-92.1 | 200.710/189.706 |
|  | CK2_S1 | *Qchl.saw-4B.2* | 4B | 2.68 | 4.24 | -0.59 | 4B_37696493 | 4B_428180567 | 33.1-40.7 | 37.696/428.180 |
|  | CK2_S1 | *Qchl.saw-4D.1* | 4D | 8.93 | 15.74 | -1.14 | 4D_101443068 | 4D_98078212 | 26.6-29.3 | 101.443/98.078 |
|  | MP1_S1 | *Qchl.saw-2A.1* | 2A | 3.87 | 6.08 | -0.75 | 2A_734756744 | 2A_704055774 | 58.3-79.4 | 734.757/704.056 |
|  | MP1_S1 | *Qchl.saw-2B.3* | 2B | 4.25 | 5.86 | -0.72 | 2B_75613720 | 2B_57119026 | 122.0-129.2 | 75.614/57.119 |
|  | MP1_S1 | *Qchl.saw-2B.4* | 2B | 2.82 | 3.95 | -0.59 | 2B_54768734 | 2B_41958287 | 131.1-137.6 | 54.769/41.958 |
|  | MP1_S1 | *Qchl.saw-4B.3* | 4B | 2.75 | 3.88 | -0.61 | 4B_628646285 | 4B_649491388 | 68.9-87.3 | 628.646/649.491 |
|  | MP1_S1 | *Qchl.saw-4D.2* | 4D | 4.40 | 7.36 | -1.17 | 4D_65859359 | 4D_15772687 | 34.4-45.9 | 65.859/15.773 |
|  | MP2_S1 | *Qchl.saw-2B.1* | 2B | 3.99 | 5.74 | -0.72 | 2B_200710374 | 2B_189706341 | 88.6-92.6 | 200.710/189.706 |
|  | MP2_S1 | *Qchl.saw-3B.1* | 3B | 4.37 | 6.76 | 0.79 | 3B_733329204 | 3B_719457438 | 62.3-63.5 | 733.329/719.457 |
|  | MP2_S1 | *Qchl.saw-3B.2* | 3B | 5.24 | 7.62 | 0.83 | 3B_707640336 | 3B_676285835 | 74.4-76.6 | 707.640/676.286 |
|  | MP2_S1 | *Qchl.saw-4D.1* | 4D | 7.51 | 11.90 | -1.08 | 4D_101443068 | 4D_98078212 | 26.6-28.9 | 101.443/98.078 |
|  | MP2_S1 | *Qchl.saw-4D.2* | 4D | 10.32 | 17.35 | -1.31 | 4D_28547729 | 4D_15772687 | 38.9-44.7 | 28.548/15.773 |
|  | MP2_S1 | *Qchl.saw-5A.1* | 5A | 3.64 | 7.96 | 0.86 | 5A_561264965 | 5A_540370129 | 144.3-164.8 | 561.265/540.370 |
|  | MP2_S1 | *Qchl.saw-7A.1* | 7A | 4.64 | 6.72 | -0.79 | 7A_67672049 | 7A_49701013 | 86.1-96.5 | 67.672/49.701 |
|  | LP1_S1 | *Qchl.saw-2B.5* | 2B | 5.82 | 9.03 | -0.84 | 2B_667125416 | 2B_661454401 | 26.6-28.4 | 667.125/661.454 |
|  | LP1_S1 | *Qchl.saw-2B.6* | 2B | 5.16 | 8.09 | -0.79 | 2B_635151521 | 2B_605628346 | 33.2-36.8 | 635.152/605.628 |
|  | LP1_S1 | *Qchl.saw-3B.1* | 3B | 2.81 | 3.95 | 0.56 | 3B_738758982 | 3B_719457438 | 60.4-63.5 | 738.758/719.457 |
|  | LP1_S1 | *Qchl.saw-3B.3* | 3B | 3.97 | 5.74 | 0.67 | 3B_676285806 | 3B_581073077 | 78.8-87.2 | 676.286/581.073 |
|  | LP1_S1 | *Qchl.saw-3B.4* | 3B | 3.40 | 4.74 | 0.61 | 3B_586733623 | 3B_561972756 | 87.8-92.1 | 586.734/561.973 |
|  | LP1_S1 | *Qchl.saw-3B.5* | 3B | 3.69 | 5.14 | 0.63 | 3B_592271369 | 3B_649898939 | 99.3-105.1 | 592.271/649.899 |
|  | LP1_S1 | *Qchl.saw-3B.6* | 3B | 2.87 | 4.03 | 0.56 | 3B_649898939 | 3B_560274100 | 105.6-113 | 649.899/560.274 |
|  | LP1_S1 | *Qchl.saw-3B.7* | 3B | 2.58 | 3.64 | 0.53 | 3B_244023572 | 3B_247207394 | 130.5-134.1 | 244.024/247.207 |
|  | LP1_S1 | *Qchl.saw-3B.8* | 3B | 3.22 | 4.51 | 0.59 | 3B_66194730 | 3B_60880348 | 164.7-168.3 | 66.195/60.880 |
|  | LP1_S1 | *Qchl.saw-4B.4* | 4B | 2.62 | 3.97 | -0.55 | 4B_515175803 | 4B_500399496 | 47.2-51.4 | 515.176/500.400 |
|  | LP1_S1 | *Qchl.saw-4B.5* | 4B | 3.20 | 4.82 | -0.61 | 4B_570246805 | 4B_604888524 | 56.9-62.5 | 570.247/604.889 |
|  | LP1_S1 | *Qchl.saw-4B.6* | 4B | 3.09 | 5.01 | -0.62 | 4B_604888524 | 4B_621946172 | 62.5-66.9 | 604.889/621.946 |
|  | LP1_S1 | *Qchl.saw-4D.1* | 4D | 7.18 | 12.04 | -0.96 | 4D_101443068 | 4D_98078212 | 27.2-29.9 | 101.443/98.078 |
|  | LP1_S1 | *Qchl.saw-4D.2* | 4D | 10.16 | 17.79 | -1.17 | 4D_28547729 | 4D_15772687 | 38.9-45.9 | 28.548/15.773 |
|  | LP1_S1 | *Qchl.saw-7A.2* | 7A | 2.70 | 4.76 | -0.61 | 7A_47338513 | 7A_36181599 | 105.0-123.0 | 47.339/36.182 |
|  | LP2_S1 | *Qchl.saw-2B.7* | 2B | 2.76 | 3.82 | 0.61 | 2B_31636243 | 2B_18638491 | 156.8-171.1 | 31.636/18.638 |
|  | LP2_S1 | *Qchl.saw-3B.9* | 3B | 3.60 | 6.11 | 0.69 | 3B_767284492 | 3B_761465652 | 29.4-44.7 | 767.284/761.466 |
|  | LP2_S1 | *Qchl.saw-4B.7* | 4B | 3.70 | 5.06 | -0.64 | 4B_28739948 | 4B_37696493 | 21.9-37.2 | 28.740/37.696 |
|  | LP2_S1 | *Qchl.saw-4B.2* | 4B | 4.38 | 5.94 | -0.70 | 4B_277277994 | 4B_433456547 | 37.2-39.7 | 277.277/433.457 |
|  | LP2_S1 | *Qchl.saw-4B.8* | 4B | 5.11 | 6.88 | -0.75 | 4B_515175803 | 4B_500399496 | 47.8-51.8 | 515.176/500.400 |
|  | LP2_S1 | *Qchl.saw-4B.9* | 4B | 4.58 | 6.34 | -0.70 | 4B_539005009 | 4B_618329284 | 55.6-64.7 | 539.005/618.329 |
|  | LP2_S1 | *Qchl.saw-4D.1* | 4D | 14.66 | 27.31 | -1.46 | 4D_101443068 | 4D_98078212 | 26.6-29.0 | 101.443/98.078 |
|  | LP2_S1 | *Qchl.saw-4D.2* | 4D | 17.26 | 31.66 | -1.59 | 4D_28547729 | 4D_15772687 | 38.9-43.4 | 28.548/15.773 |
|  | LP2_S1 | *Qchl.saw-5A.2* | 5A | 3.46 | 5.14 | -0.62 | 5A_15723623 | 5A_9510725 | 300.9-317.7 | 15.724/9.511 |
|  | BLUP_S1 | *Qchl.saw-2B.1* | 2B | 7.05 | 11.63 | -0.83 | 2B_162775090 | 2B_189706341 | 88.6-92.6 | 162.775/189.706 |
|  | BLUP_S1 | *Qchl.saw-2B.8* | 2B | 4.96 | 5.75 | -0.45 | 2B_41958287 | 2B_41958337 | 136.6-137.9 | 41.958/41.958 |
|  | BLUP_S1 | *Qchl.saw-2D.1* | 2D | 3.04 | 9.54 | 1.58 | 2D_601107080 | 2D_599099549 | 1.0-3.0 | 601.107/599.100 |
|  | BLUP_S1 | *Qchl.saw-3B.10* | 3B | 9.90 | 9.98 | 0.61 | 3B_774883534 | 3B_767284492 | 29.1-34.4 | 774.884/767.284 |
|  | BLUP_S1 | *Qchl.saw-3B.9* | 3B | 9.68 | 9.93 | 0.61 | 3B_767284492 | 3B_761465652 | 35.3-44.9 | 767.284/761.466 |
|  | BLUP_S1 | *Qchl.saw-3B.11* | 3B | 9.05 | 8.53 | 0.57 | 3B_751013879 | 3B_749137398 | 49.6-52.5 | 751.014/749.137 |
|  | BLUP_S1 | *Qchl.saw-3B.1* | 3B | 10.99 | 10.14 | 0.61 | 3B_736664044 | 3B_719457438 | 61.0-63.2 | 736.664/719.457 |
|  | BLUP_S1 | *Qchl.saw-3B.2* | 3B | 12.53 | 11.36 | 0.64 | 3B_707640336 | 3B_676285835 | 74.6-76.7 | 707.640/676.286 |
|  | BLUP_S1 | *Qchl.saw-3B.4* | 3B | 8.52 | 8.08 | 0.54 | 3B_586733623 | 3B_561972756 | 87.8-91.1 | 586.734/561.973 |
|  | BLUP_S1 | *Qchl.saw-3B.5* | 3B | 9.91 | 9.54 | 0.59 | 3B_592271369 | 3B_649898939 | 99.3-105.1 | 592.271/649.899 |
|  | BLUP_S1 | *Qchl.saw-3B.12* | 3B | 7.11 | 7.06 | 0.51 | 3B_620204195 | 3B_595671050 | 108.5-111.0 | 620.204/595.671 |
|  | BLUP_S1 | *Qchl.saw-3B.13* | 3B | 5.84 | 5.82 | 0.45 | 3B_508486793 | 3B_437601469 | 119.3-121.5 | 508.487/437.601 |
|  | BLUP_S1 | *Qchl.saw-3B.7* | 3B | 5.65 | 5.53 | 0.44 | 3B_244023572 | 3B_247207394 | 131.1-133.6 | 244.024/247.207 |
|  | BLUP_S1 | *Qchl.saw-3B.14* | 3B | 4.74 | 4.69 | 0.41 | 3B_445338984 | 3B_260343602 | 140.6-148.0 | 445.339/260.344 |
|  | BLUP_S1 | *Qchl.saw-3B.15* | 3B | 4.91 | 4.85 | 0.41 | 3B_344296760 | 3B_217452695 | 153.2-155.5 | 344.297/217.453 |
|  | BLUP_S1 | *Qchl.saw-3B.8* | 3B | 5.92 | 5.82 | 0.45 | 3B_66194730 | 3B_60880348 | 164.4-168.3 | 66.195/60.880 |
|  | BLUP_S1 | *Qchl.saw-4B.2* | 4B | 3.28 | 7.26 | 1.41 | 4B_454527626 | 4B_433456547 | 37.8-39.7 | 454.528/433.457 |
|  | BLUP_S1 | *Qchl.saw-4B.10* | 4B | 3.93 | 3.59 | 0.36 | 4B_427062234 | 4B_515175803 | 41.1-47.8 | 427.062/515.176 |
|  | BLUP_S1 | *Qchl.saw-4D.1* | 4D | 3.42 | 3.07 | -0.33 | 4D_101443068 | 4D_98078212 | 26.6-29.0 | 101.443/98.078 |
|  | BLUP_S1 | *Qchl.saw-4D.3* | 4D | 4.12 | 5.12 | -0.53 | 4D_48697668 | 4D_28547729 | 36.5-38.8 | 48.698/28.548 |
|  | BLUP_S1 | *Qchl.saw-5A.3* | 5A | 3.07 | 10.41 | -0.45 | 5A_636697253 | 5A_613546850 | 70.5-84.2 | 636.697/613.547 |
|  | BLUP_S1 | *Qchl.saw-5A.4* | 5A | 3.04 | 7.18 | 1.40 | 5A_572980094 | 5A_572006166 | 129-130.9 | 572.980/572.006 |
| S2 | CK1_S2 | *Qchl.saw-2A.2* | 2A | 4.27 | 6.32 | -0.68 | 2A_726132029 | 2A_704892430 | 73.9-81.0 | 726.132/704.892 |
|  | CK1_S2 | *Qchl.saw-2A.3* | 2A | 4.28 | 6.33 | -0.68 | 2A_702061597 | 2A_690534124 | 85.7-90.5 | 702.062/690.534 |
|  | CK1_S2 | *Qchl.saw-2B.1* | 2B | 4.44 | 6.57 | -0.71 | 2B_200710374 | 2B_189706341 | 88.4-91.2 | 200.710/189.706 |
|  | CK1_S2 | *Qchl.saw-2B.9* | 2B | 4.02 | 5.99 | -0.68 | 2B_151463186 | 2B_158445765 | 96.7-99.9 | 151.463/158.446 |
|  | CK1_S2 | *Qchl.saw-3B.16* | 3B | 4.23 | 9.42 | 0.87 | 3B_784079362 | 3B_774883534 | 14.3-29.4 | 784.079/774.884 |
|  | CK1_S2 | *Qchl.saw-3B.17* | 3B | 3.81 | 5.74 | 0.68 | 3B_774883534 | 3B_764742114 | 29.4-42.9 | 774.884/764.742 |
|  | CK1_S2 | *Qchl.saw-4D.4* | 4D | 10.46 | 17.85 | -1.19 | 4D_184747096 | 4D_317656896 | 24.1-28.4 | 184.747/317.657 |
|  | CK1_S2 | *Qchl.saw-4D.3* | 4D | 12.66 | 21.30 | -1.30 | 4D_48697668 | 4D_28547729 | 33.4-38.9 | 48.698/28.548 |
|  | CK1_S2 | *Qchl.saw-4D.2* | 4D | 12.84 | 22.62 | -1.34 | 4D_28547729 | 4D_15772687 | 38.9-45.9 | 28.548/15.773 |
|  | CK1_S2 | *Qchl.saw-5A.5* | 5A | 2.93 | 4.47 | 0.58 | 5A_636697253 | 5A_613546850 | 72.5-93.5 | 636.697/613.547 |
|  | CK2_S2 | *Qchl.saw-3B.18* | 3B | 2.92 | 5.47 | -1.51 | 3B_750131708 | 3B_748417876 | 50.6-54.1 | 750.132/748.418 |
|  | CK2_S2 | *Qchl.saw-3B.19* | 3B | 4.17 | 6.86 | -2.18 | 3B_738761356 | 3B_736664044 | 59-61.3 | 738.761/736.664 |
|  | CK2_S2 | *Qchl.saw-3B.2* | 3B | 8.18 | 15.12 | 3.18 | 3B_707640336 | 3B_683057859 | 74.4-75.6 | 707.640/683.057 |
|  | CK2_S2 | *Qchl.saw-4A.1* | 4A | 3.48 | 6.38 | -0.71 | 4A_648910616 | 4A_672508553 | 59.6-73.1 | 648.911/672.509 |
|  | MP1_S2 | *Qchl.saw-4D.5* | 4D | 2.78 | 5.69 | -0.64 | 4D_433505595 | 4D_403202323 | 9.4-19.5 | 433.506/403.202 |
|  | MP1_S2 | *Qchl.saw-4D.4* | 4D | 3.16 | 6.13 | -0.66 | 4D_184747096 | 4D_317656896 | 20.1-29.7 | 184.747/317.657 |
|  | MP1_S2 | *Qchl.saw-7A.3* | 7A | 2.72 | 5.30 | -0.62 | 7A_92745962 | 7A_84244400 | 69.4-77.7 | 92.746/84.244 |
|  | MP2_S2 | *Qchl.saw-4D.1* | 4D | 6.12 | 11.13 | -1.05 | 4D_101443068 | 4D_98078212 | 27.2-29.9 | 101.443/98.078 |
|  | MP2_S2 | *Qchl.saw-4D.2* | 4D | 8.99 | 15.83 | -1.25 | 4D_48697668 | 4D_15772687 | 36.2-43.6 | 48.697/15.773 |
|  | MP2_S2 | *Qchl.saw-5A.6* | 5A | 2.78 | 7.21 | 0.84 | 5A_558969437 | 5A_540370129 | 146.7-169.2 | 558.970/540.370 |
|  | LP1_S2 | *Qchl.saw-2A.4* | 2A | 2.63 | 4.45 | -0.62 | 2A_738029301 | 2A_704055774 | 55.1-79.5 | 738.029/704.0558 |
|  | LP1_S2 | *Qchl.saw-2B.10* | 2B | 4.60 | 6.85 | -0.77 | 2B_207149824 | 2B_192389178 | 87.9-91.2 | 207.150/192.390 |
|  | LP1_S2 | *Qchl.saw-4D.1* | 4D | 8.26 | 14.54 | -1.13 | 4D_101443068 | 4D_98078212 | 27.2-29.9 | 101.443/98.078 |
|  | LP1_S2 | *Qchl.saw-4D.6* | 4D | 10.19 | 17.00 | -1.22 | 4D_61613658 | 4D_48697668 | 32.9-35.9 | 61.614/48.698 |
|  | LP1_S2 | *Qchl.saw-4D.2* | 4D | 12.18 | 19.87 | -1.33 | 4D_28547729 | 4D_15772687 | 38.8-42.4 | 28.548/15.773 |
|  | LP1_S2 | *Qchl.saw-5A.7* | 5A | 2.71 | 3.96 | 0.59 | 5A_591302327 | 5A_572980094 | 111.9-127.7 | 591.302/572.980 |
|  | LP2_S2 | *Qchl.saw-3B.1* | 3B | 2.81 | 4.99 | 0.67 | 3B_736664044 | 3B_724746254 | 61.0-65.0 | 736.664/724.746 |
|  | LP2_S2 | *Qchl.saw-3B.20* | 3B | 3.33 | 5.87 | 0.73 | 3B_722455022 | 3B_732314158 | 68.4-73.5 | 722.455/732.314 |
|  | LP2_S2 | *Qchl.saw-4D.1* | 4D | 5.14 | 9.80 | -0.96 | 4D_101443068 | 4D_98078212 | 27.2-29.3 | 101.443/98.078 |
|  | LP2_S2 | *Qchl.saw-4D.2* | 4D | 7.53 | 13.96 | -1.14 | 4D_28547729 | 4D_15772687 | 34.4-43.1 | 28.548/15.773 |
|  | LP2_S2 | *Qchl.saw-5A.8* | 5A | 2.81 | 7.29 | 0.81 | 5A_666212352 | 5A_645253609 | 48.6-66.6 | 666.212/645.254 |
|  | LP2_S2 | *Qchl.saw-5A.9* | 5A | 3.87 | 7.43 | 0.81 | 5A_645253609 | 5A_613546850 | 67.8-81.9 | 645.254/613.547 |
|  | LP2_S2 | *Qchl.saw-7B.1* | 7B | 2.75 | 4.53 | 0.64 | 7B_659732599 | 7B_633102498 | 5.5-11.1 | 659.732/633.102 |
|  | BLUP_S2 | *Qchl.saw-2B.1* | 2B | 5.23 | 9.07 | 1.54 | 2B_199236813 | 2B_189706341 | 89.2-92.1 | 199.236/189.706 |
|  | BLUP_S2 | *Qchl.saw-4A.2* | 4A | 2.52 | 5.72 | 0.46 | 4A_713513570 | 4A_704648755 | 16.1-28.4 | 713.513/704.649 |
|  | BLUP_S2 | *Qchl.saw-4A.3* | 4A | 4.07 | 3.59 | -0.36 | 4A_700964251 | 4A_690657706 | 36.0-51.2 | 700.964/690.658 |
|  | BLUP_S2 | *Qchl.saw-4A.4* | 4A | 3.69 | 4.28 | -0.39 | 4A_644613024 | 4A_666811268 | 58.9-67.5 | 644.613/666.811 |
|  | BLUP_S2 | *Qchl.saw-4A.5* | 4A | 2.77 | 4.11 | -0.28 | 4A_672508702 | 4A_638262359 | 79.8-96.4 | 672.509/638.262 |
|  | BLUP_S2 | *Qchl.saw-4A.6* | 4A | 2.64 | 8.77 | -0.41 | 4A_635943789 | 4A_620278986 | 105.4-118 | 635.944/620.279 |
|  | BLUP_S2 | *Qchl.saw-4D.4* | 4D | 5.80 | 6.67 | -0.36 | 4D_184747096 | 4D_317656896 | 25.1-27.5 | 184.747/317.657 |
|  | BLUP_S2 | *Qchl.saw-4D.2* | 4D | 7.67 | 4.48 | -0.29 | 4D_48697668 | 4D_28547729 | 36.7-41.1 | 48.697/28.547 |
|  | BLUP_S2 | *Qchl.saw-6A.1* | 6A | 2.55 | 9.14 | -0.58 | 6A_614543947 | 6A_609309941 | 0.0-5.0 | 614.544/609.310 |
|  | BLUP_S2 | *Qchl.saw-7A.4* | 7A | 3.21 | 4.48 | -0.30 | 7A_15677256 | 7A_6846747 | 0.0-8.1 | 15.677/6.847 |
| S3 | CK1_S3 | *Qchl.saw-4B.4* | 4B | 3.08 | 5.88 | -1.05 | 4B_515175803 | 4B_500399496 | 43.0-53.9 | 515.176/500.400 |
|  | CK1_S3 | *Qchl.saw-4B.11* | 4B | 3.35 | 6.38 | -1.11 | 4B_612486206 | 4B_628646285 | 63.0-68.6 | 612.486/628.646 |
|  | CK1_S3 | *Qchl.saw-5A.9* | 5A | 2.60 | 5.30 | 0.85 | 5A_645253609 | 5A_613546850 | 67.1-83.6 | 645.254/613.547 |
|  | CK2_S3 | *Qchl.saw-2B.11* | 2B | 3.28 | 6.41 | -0.75 | 2B_297999025 | 2B_192389178 | 82.3-91.2 | 297.999/192.389 |
|  | CK2_S3 | *Qchl.saw-6B.1* | 6B | 3.85 | 7.53 | 0.82 | 6B_676534940 | 6B_687067128 | 127.3-143.4 | 676.535/687.067 |
|  | MP1_S3 | *Qchl.saw-7A.5* | 7A | 2.62 | 5.38 | -1.19 | 7A_191261406 | 7A_101617032 | 52.9-66.2 | 191.261/101.617 |
|  | MP2_S3 | *Qchl.saw-4D.3* | 4D | 3.49 | 8.13 | -0.94 | 4D_48697668 | 4D_28547729 | 34.1-45.9 | 48.698/28.548 |
|  | MP2_S3 | *Qchl.saw-5A.9* | 5A | 2.95 | 6.15 | 0.82 | 5A_645253609 | 5A_613546850 | 65.8-83.3 | 645.254/613.547 |
|  | LP1_S3 | *Qchl.saw-5D* | 5D | 2.59 | 4.85 | 2.37 | 5D_390409478 | 5D_308355138 | 0.0-18.3 | 390.409/308.355 |
|  | LP1_S3 | *Qchl.saw-6A.2* | 6A | 2.73 | 5.10 | 2.46 | 6A_61743025 | 6A_42008503 | 52.2-68.8 | 61.743/42.009 |
|  | LP1_S3 | *Qchl.saw-6B.2* | 6B | 3.30 | 6.14 | -6.22 | 6B_655865257 | 6B_659573035 | 115.6-118.2 | 655.865/659.573 |
|  | LP1_S3 | *Qchl.saw-6B.3* | 6B | 3.58 | 6.63 | -4.21 | 6B_668891452 | 6B_681412567 | 121.6-132.3 | 668.891/681.413 |
|  | LP2_S3 | *Qchl.saw-4A.7* | 4A | 2.53 | 4.63 | -1.53 | 4A_707934625 | 4A_690657706 | 20.8-44.1 | 707.935/690.658 |
|  | LP2_S3 | *Qchl.saw-5A.9* | 5A | 3.10 | 5.88 | 1.71 | 5A_645253609 | 5A_613546850 | 64.8-82.0 | 645.254/613.547 |
|  | BLUP_S3 | *Qchl.saw-4A.8* | 4A | 3.64 | 7.57 | -1.41 | 4A_704648755 | 4A_690657706 | 30.6-51.2 | 704.649/690.658 |
|  | BLUP_S3 | *Qchl.saw-4A.5* | 4A | 4.01 | 3.55 | -1.08 | 4A_672508702 | 4A_638262359 | 79.8-96.1 | 672.509/638.262 |
|  | BLUP_S3 | *Qchl.saw-4A.9* | 4A | 2.94 | 4.32 | -1.23 | 4A_639942192 | 4A_628023767 | 98.8-109.4 | 639.942/628.023 |
|  | BLUP_S3 | *Qchl.saw-5A.9* | 5A | 6.51 | 12.60 | 2.12 | 5A_645253609 | 5A_613546850 | 66.4-83.7 | 645.254/613.547 |
|  | BLUP_S3 | *Qchl.saw-7B.2* | 7B | 2.79 | 5.66 | -0.45 | 7B_659732599 | 7B_640017468 | 4.7-9.0 | 659.733/640.017 |
| S4 | CK1_S4 | *Qchl.saw-6A.3* | 6A | 2.65 | 4.96 | 3.05 | 6A_454622177 | 6A_350196795 | 36.4-40.7 | 454.622/350.197 |
|  | CK1_S4 | *Qchl.saw-6A.4* | 6A | 2.58 | 4.83 | 2.98 | 6A_69374520 | 6A_61743025 | 49.3-52.2 | 69.375/61.743 |
|  | CK2_S4 | *Qchl.saw-4A.10* | 4A | 4.29 | 8.04 | -3.60 | 4A_683950816 | 4A_639942192 | 89.1-98.8 | 683.951/639.942 |
|  | CK2_S4 | *Qchl.saw-4A.11* | 4A | 4.40 | 7.39 | -3.43 | 4A_639942192 | 4A_628023767 | 98.8-109.1 | 639.942/628.024 |
|  | CK2_S4 | *Qchl.saw-4A.12* | 4A | 3.70 | 6.73 | -3.28 | 4A_628023767 | 4A_620278986 | 110-123.5 | 628.024/620.279 |
|  | CK2_S4 | *Qchl.saw-4B.7* | 4B | 6.26 | 10.88 | -4.18 | 4B_28739948 | 4B_37696493 | 25.9-34.1 | 28.740/37.696 |
|  | CK2_S4 | *Qchl.saw-4B.2* | 4B | 3.42 | 6.01 | -3.12 | 4B_454527626 | 4B_433456547 | 38.2-40.0 | 454.528/433.457 |
|  | MP1_S4 | *Qchl.saw-3A.3* | 3A | 3.63 | 7.18 | 3.52 | 3A_597825929 | 3A_638328149 | 132.4-137.5 | 597.826/638.328 |
|  | MP1_S4 | *Qchl.saw-3B.21* | 3B | 3.17 | 6.39 | -3.12 | 3B_247207322 | 3B_260343602 | 146.0-148.0 | 247.207/260.344 |
|  | MP1_S4 | *Qchl.saw-3B.22* | 3B | 3.46 | 6.94 | -3.07 | 3B_312733367 | 3B_256451885 | 152-153.9 | 312.733/256.452 |
|  | MP1_S4 | *Qchl.saw-3B.22* | 3B | 4.12 | 8.20 | -3.20 | 3B_246108343 | 3B_423362772 | 157.2-160.2 | 246.108/423.363 |
|  | MP1_S4 | *Qchl.saw-4D.7* | 4D | 2.67 | 5.28 | 3.02 | 4D_481390025 | 4D_433505595 | 0.3-12.0 | 481.390/433.506 |
|  | MP2_S4 | *Qchl.saw-3A.2* | 3A | 3.18 | 5.80 | 3.81 | 3A_686724449 | 3A_688580956 | 150.5-157.2 | 686.724/688.581 |
|  | MP2_S4 | *Qchl.saw-4D.2* | 4D | 6.12 | 12.68 | 5.67 | 4D_28547729 | 4D_15772687 | 40.2-45.9 | 28.548/15.773 |
|  | MP2_S4 | *Qchl.saw-5A.10* | 5A | 2.73 | 4.66 | 3.45 | 5A_467547080 | 5A_454838505 | 194.8-206.8 | 467.547/454.839 |
|  | MP2_S4 | *Qchl.saw-6A.4* | 6A | 3.22 | 5.89 | 3.89 | 6A_84693688 | 6A_69374520 | 47.1-49.3 | 84.693/69.375 |
|  | LP1_S4 | *Qchl.saw-1D.1* | 1D | 3.09 | 5.80 | 1.81 | 1D_340550117 | 1D_75547610 | 18.5-34.2 | 340.550/75.548 |
|  | LP1_S4 | *Qchl.saw-1D.2* | 1D | 2.77 | 5.22 | 1.72 | 1D_75547610 | 1D_37948732 | 34.2-37.1 | 75.548/37.949 |
|  | LP1_S4 | *Qchl.saw-4A.9* | 4A | 3.83 | 7.25 | -2.02 | 4A_639942192 | 4A_635943789 | 99.6-106.5 | 639.942/635.944 |
|  | LP2_S4 | *Qchl.saw-2D.2* | 2D | 2.90 | 5.10 | 2.45 | 2D_62297218 | 2D_49079010 | 65.5-73.6 | 62.297/49.079 |
|  | LP2_S4 | *Qchl.saw-4A.13* | 4A | 3.09 | 5.43 | -2.54 | 4A_672508702 | 4A_638262359 | 80.0-93.5 | 672.509/638.262 |
|  | BLUP_S4 | *Qchl.saw-3A.4* | 3A | 5.61 | 4.42 | 1.08 | 3A_622695831 | 3A_658657616 | 140.0-143.5 | 622.696/658.658 |
|  | BLUP_S4 | *Qchl.saw-3A.5* | 3A | 5.29 | 3.07 | -0.33 | 3A_650566328 | 3A_653919337 | 145.9-148.3 | 650.566/653.919 |
|  | BLUP_S4 | *Qchl.saw-4A.14* | 4A | 4.23 | 5.19 | -0.31 | 4A_672508702 | 4A_683950816 | 80.4-89.3 | 672.509683.951 |
|  | BLUP_S4 | *Qchl.saw-4D.2* | 4D | 2.63 | 4.14 | -0.29 | 4D_28547729 | 4D_15772687 | 39.7-46.9 | 28.548/15.773 |
|  | BLUP_S4 | *Qchl.saw-6A.5* | 6A | 4.28 | 9.14 | -0.67 | 6A_455986879 | 6A_350196795 | 31.0-40.7 | 455.987/350.197 |
|  | BLUP_S4 | *Qchl.saw-6A.4* | 6A | 4.33 | 11.27 | 4.70 | 6A_69374520 | 6A_64071530 | 49.3-51.7 | 69.375/64.071 |

**Table S7.** Additive effects of the four major QTLs at the S1 stage.

| *Qchl.saw-4D.1* | *Qchl.saw-4D.2* | *Qchl.saw-2B.1* | *Qchl.saw-3B.1* | Sample Size | CHL | Difference | Percent (%) |
| --- | --- | --- | --- | --- | --- | --- | --- |
| + | + | + | + | 18 | 57.37±1.51a | 4.47 | 8.45 |
| + | + | + | - | 21 | 56.48±1.14ab | 3.58 | 6.77 |
| - | + | + | + | 3 | 56.15±0.49ab | 3.25 | 6.14 |
| + | + | - | + | 12 | 56.14±1.04ab | 3.24 | 6.12 |
| + | + | - | - | 26 | 55.41±1.38bc | 2.51 | 4.74 |
| + | - | + | + | 2 | 55.30±0.14bc | 2.40 | 4.54 |
| - | + | + | - | 1 | 54.89±0.00c | 1.99 | 3.76 |
| - | + | - | + | 1 | 54.58±0.00c | 1.68 | 3.18 |
| - | - | + | + | 25 | 54.40±1.03cd | 1.50 | 2.84 |
| + | - | - | - | 2 | 54.10±0.37cd | 1.20 | 2.27 |
| - | - | - | + | 35 | 53.92±1.16cd | 1.02 | 1.93 |
| - | - | + | - | 20 | 53.84±1.10cd | 0.94 | 1.78 |
| + | - | + | - | 2 | 53.71±3.62cd | 0.81 | 1.53 |
| - | + | - | - | 1 | 53.62±0.00d | 0.72 | 1.36 |
| + | - | - | + | 4 | 53.34±0.71d | 0.44 | 0.83 |
| - | - | - | - | 25 | 52.90±1.29d | 0.00 | 0.00 |

“+” and “-” represent lines with and without the positive alleles of the target quantitative trait loci based on the flanking markers of the corresponding QTL.

**Table S8. Functional annotation and enrichment of genes of the six major QTLs.**

| Chr. | Start | Stop | Ori | Name | Function |
| --- | --- | --- | --- | --- | --- |
| chr2B | 162927851 | 162929768 | - | *TraesCS2B02G187600* | integral component of membrane, lipid binding, lipid transport |
| chr2B | 164088025 | 164091566 | - | *TraesCS2B02G188600* | nucleic acid binding |
| chr2B | 164638487 | 164642697 | + | *TraesCS2B02G189000* | endoplasmic reticulum membrane |
| chr2B | 164936500 | 164940359 | + | *TraesCS2B02G189300* | chloroplast |
| chr2B | 165115340 | 165119793 | - | *TraesCS2B02G189600* | ATP binding, protein phosphorylation |
| chr2B | 165244218 | 165246318 | + | *TraesCS2B02G189700* | oxidoreductase activity |
| chr2B | 165250391 | 165253311 | + | *TraesCS2B02G189800* | oxidoreductase activity |
| chr2B | 165503040 | 165512693 | + | *TraesCS2B02G190100* | nucleic acid binding, ATP binding |
| chr2B | 166210291 | 166210606 | + | *TraesCS2B02G190600* | photosynthesis, light reaction |
| chr2B | 166466357 | 166466468 | - | *TraesCS2B02G191000* | integral component of membrane |
| chr2B | 166468399 | 166471957 | + | *TraesCS2B02G191100* | GTP binding |
| chr2B | 167081259 | 167086767 | + | *TraesCS2B02G191800* | ATP binding, protein phosphorylation |
| chr2B | 168620692 | 168623229 | - | *TraesCS2B02G192900* | cellular metabolic process |
| chr2B | 168623648 | 168630147 | - | *TraesCS2B02G193000* | ATP binding, protein phosphorylation |
| chr2B | 169864359 | 169865163 | + | *TraesCS2B02G193500* | integral component of membrane |
| chr2B | 170388231 | 170393777 | + | *TraesCS2B02G193600* | pyridoxal phosphate binding, biosynthetic process |
| chr2B | 170851542 | 170852486 | - | *TraesCS2B02G193800* | integral component of membrane |
| chr2B | 171461107 | 171466379 | - | *TraesCS2B02G194600* | oxidoreductase activity |
| chr2B | 172218741 | 172224976 | + | *TraesCS2B02G195100* | nucleic acid binding |
| chr2B | 172672489 | 172679370 | + | *TraesCS2B02G195200* | nucleic acid binding |
| chr2B | 172890190 | 172895217 | - | *TraesCS2B02G195300* | nucleic acid binding |
| chr2B | 173485105 | 173486277 | + | *TraesCS2B02G195900* | cellular response to phosphate starvation |
| chr2B | 173754889 | 173757421 | - | *TraesCS2B02G196000* | integral component of membrane |
| chr2B | 174591804 | 174593791 | - | *TraesCS2B02G196500* | biosynthetic process |
| chr2B | 174792309 | 174799265 | - | *TraesCS2B02G196800* | nucleic acid binding |
| chr2B | 174827893 | 174831599 | - | *TraesCS2B02G197000* | ATP binding, protein phosphorylation |
| chr2B | 174923314 | 174932558 | - | *TraesCS2B02G197100* | small GTPase binding |
| chr2B | 175454755 | 175455112 | + | *TraesCS2B02G197700* | integral component of membrane |
| chr2B | 175638456 | 175649555 | - | *TraesCS2B02G198300* | ATP binding |
| chr2B | 177218039 | 177218459 | - | *TraesCS2B02G199100* | integral component of membrane |
| chr2B | 178699868 | 178711584 | - | *TraesCS2B02G199900* | ATP binding |
| chr2B | 178723854 | 178724994 | + | *TraesCS2B02G200000* | nucleobase-containing compound metabolic process |
| chr2B | 178857025 | 178857786 | - | *TraesCS2B02G200400* | integral component of membrane |
| chr2B | 180436516 | 180437318 | - | *TraesCS2B02G201000* | integral component of membrane |
| chr2B | 180616691 | 180619271 | - | *TraesCS2B02G201700* | carbohydrate metabolic process, pentose-phosphate shunt |
| chr2B | 182355758 | 182361233 | + | *TraesCS2B02G202600* | integral component of membrane |
| chr2B | 182705866 | 182707578 | + | *TraesCS2B02G202800* | phosphoric diester hydrolase activity |
| chr2B | 182708241 | 182711907 | - | *TraesCS2B02G202900* | ATP binding, protein phosphorylation |
| chr2B | 183211066 | 183212793 | + | *TraesCS2B02G203600* | integral component of membrane |
| chr2B | 184133485 | 184135102 | - | *TraesCS2B02G204300* | carbohydrate metabolic process |
| chr2B | 184326362 | 184332008 | + | *TraesCS2B02G204500* | oxidoreductase activity, acting on the CH-OH group of donors, NAD or NADP as acceptor |
| chr2B | 184955705 | 184961151 | - | *TraesCS2B02G206300* | nucleotidyltransferase activity |
| chr2B | 185800521 | 185804495 | + | *TraesCS2B02G206900* | ADP binding |
| chr2B | 189616940 | 189632226 | - | *TraesCS2B02G208400* | nucleic acid binding, ATP binding |
| chr3B | 720252251 | 720256185 | + | *TraesCS3B02G471800* | ADP binding |
| chr3B | 720889906 | 720893772 | - | *TraesCS3B02G472100* | ATP binding, phosphatidylinositol phosphate kinase activity |
| chr3B | 720957139 | 720959888 | + | *TraesCS3B02G472200* | carbohydrate binding, protein phosphorylation |
| chr3B | 721037153 | 721042091 | + | *TraesCS3B02G472500* | ADP binding |
| chr3B | 722359091 | 722363620 | - | *TraesCS3B02G473100* | ATP binding, protein phosphorylation |
| chr3B | 722365160 | 722366828 | + | *TraesCS3B02G473200* | nucleic acid binding |
| chr3B | 722475646 | 722480010 | - | *TraesCS3B02G473300* | ATP binding |
| chr3B | 723031626 | 723035032 | - | *TraesCS3B02G473800* | pentose-phosphate shunt, chloroplast stroma, carbohydrate metabolic process |
| chr3B | 723150024 | 723154204 | - | *TraesCS3B02G474000* | integral component of membrane |
| chr3B | 723456693 | 723460964 | + | *TraesCS3B02G474400* | ATP binding |
| chr3B | 723463619 | 723467882 | + | *TraesCS3B02G474500* | ATP binding |
| chr3B | 723505378 | 723508545 | - | *TraesCS3B02G474900* | integral component of membrane |
| chr3B | 724182298 | 724185871 | + | *TraesCS3B02G475600* | ATP binding |
| chr3B | 724750461 | 724755189 | - | *TraesCS3B02G475900* | ATP binding, phosphorylation |
| chr3B | 725387951 | 725392494 | + | *TraesCS3B02G476800* | phosphatase inhibitor activity |
| chr3B | 725565633 | 725573936 | - | *TraesCS3B02G477000* | pyridoxal phosphate binding |
| chr3B | 725577635 | 725581339 | - | *TraesCS3B02G477100* | pyridoxal phosphate binding, biosynthetic process |
| chr3B | 725707258 | 725708766 | + | *TraesCS3B02G477300* | chloroplast thylakoid membrane, chloroplast |
| chr3B | 725708564 | 725712289 | - | *TraesCS3B02G477400* | ATP binding, protein phosphorylation |
| chr3B | 726441011 | 726442854 | - | *TraesCS3B02G477700* | oxidoreductase activity, acting on the CH-OH group of donors, NAD or NADP as acceptor |
| chr3B | 726480645 | 726488658 | - | *TraesCS3B02G478000* | ATP binding |
| chr3B | 726770458 | 726780334 | - | *TraesCS3B02G478400* | phosphorylation |
| chr3B | 727156826 | 727159611 | + | *TraesCS3B02G478800* | carbohydrate binding |
| chr3B | 727238217 | 727245658 | - | *TraesCS3B02G478900* | ATP binding |
| chr3B | 727316194 | 727323690 | - | *TraesCS3B02G479100* | ATP binding, phosphorylation |
| chr3B | 728809469 | 728813034 | - | *TraesCS3B02G481000* | ATP binding |
| chr3B | 729227727 | 729228995 | + | *TraesCS3B02G481900* | nucleic acid phosphodiester bond hydrolysis |
| chr3B | 729377319 | 729387298 | + | *TraesCS3B02G482000* | integral component of membrane |
| chr3B | 729414681 | 729415980 | + | *TraesCS3B02G482200* | ATP binding,protein phosphorylation |
| chr3B | 729804137 | 729805277 | + | *TraesCS3B02G482900* | ATP binding,protein phosphorylation |
| chr3B | 729920914 | 729922022 | - | *TraesCS3B02G483000* | ATP binding,protein phosphorylation |
| chr3B | 730281956 | 730286418 | + | *TraesCS3B02G483600* | ADP binding |
| chr3B | 730308538 | 730312205 | + | *TraesCS3B02G483700* | ADP binding |
| chr3B | 730313099 | 730314236 | + | *TraesCS3B02G483800* | carbohydrate metabolic process |
| chr3B | 730314625 | 730316434 | + | *TraesCS3B02G483900* | carbohydrate metabolic process |
| chr3B | 730342134 | 730345629 | + | *TraesCS3B02G484000* | chloroplast |
| chr3B | 730937416 | 730940225 | + | *TraesCS3B02G484500* | oxidoreductase activity, acting on the CH-OH group of donors, NAD or NADP as acceptor |
| chr3B | 731262890 | 731265718 | + | *TraesCS3B02G485000* | oxidoreductase activity |
| chr3B | 731479090 | 731487249 | + | *TraesCS3B02G485100* | integral component of membrane |
| chr3B | 731525889 | 731530482 | + | *TraesCS3B02G485200* | ADP binding |
| chr3B | 731552290 | 731556555 | + | *TraesCS3B02G485300* | ADP binding |
| chr3B | 732427297 | 732430961 | - | *TraesCS3B02G485600* | integral component of membrane |
| chr3B | 732432999 | 732436374 | - | *TraesCS3B02G485800* | phospholipid metabolic process |
| chr3B | 732438946 | 732442621 | - | *TraesCS3B02G485900* | ATP binding |
| chr3B | 733879486 | 733884400 | + | *TraesCS3B02G487900* | ATP binding, ATP hydrolysis activity |
| chr3B | 735380774 | 735381803 | - | *TraesCS3B02G489500* | regulation of flower development |
| chr3B | 736420303 | 736431741 | - | *TraesCS3B02G490000* | ATP binding |
| chr3B | 736651720 | 736659367 | + | *TraesCS3B02G490300* | ATP binding, carbohydrate binding |
| chr4D | 98441989 | 98444445 | + | *TraesCS4D02G117800* | lipid metabolic process |
| chr4D | 98447285 | 98453878 | + | *TraesCS4D02G117900* | chloroplast membrane |
| chr4D | 98944808 | 98945438 | - | *TraesCS4D02G118400* | ADP binding |
| chr4D | 99161359 | 99162709 | - | *TraesCS4D02G118500* | ATP binding |
| chr4D | 100319857 | 100320223 | + | *TraesCS4D02G118900* | lipid binding |
| chr4D | 100566427 | 100570411 | - | *TraesCS4D02G119200* | chloroplast stroma |
| chr4D | 15787866 | 15790386 | + | *TraesCS4D02G034700* | chloroplast, GTP binding |
| chr4D | 15795307 | 15797831 | + | *TraesCS4D02G034800* | chloroplast, GTP binding |
| chr4D | 15962172 | 15963076 | - | *TraesCS4D02G035400* | integral component of membrane |
| chr4D | 16006789 | 16009391 | - | *TraesCS4D02G035600* | chloroplast |
| chr4D | 16098176 | 16105037 | - | *TraesCS4D02G036000* | integral component of membrane |
| chr4D | 16146440 | 16147710 | + | *TraesCS4D02G036100* | integral component of membrane, transferase activity, lipid metabolic process |
| chr4D | 16231199 | 16235987 | - | *TraesCS4D02G036400* | phosphorelay sensor kinase activity |
| chr4D | 16244816 | 16251468 | + | *TraesCS4D02G036500* | ATP binding, phosphatidylinositol phosphate kinase activity |
| chr4D | 16331014 | 16332120 | + | *TraesCS4D02G037300* | hydrolase activity |
| chr4D | 16447198 | 16449343 | - | *TraesCS4D02G038100* | integral component of membrane |
| chr4D | 16533506 | 16539743 | + | *TraesCS4D02G038200* | integral component of membrane |
| chr4D | 16584269 | 16585255 | - | *TraesCS4D02G038400* | integral component of membrane |
| chr4D | 16585261 | 16589394 | - | *TraesCS4D02G038500* | chloroplast |
| chr4D | 16606852 | 16610842 | - | *TraesCS4D02G038600* | chloroplast, carbohydrate metabolic process |
| chr4D | 16650028 | 16654353 | - | *TraesCS4D02G038800* | integral component of membrane |
| chr4D | 16655045 | 16661666 | - | *TraesCS4D02G038900* | ATP binding |
| chr4D | 16797472 | 16799127 | + | *TraesCS4D02G039100* | nucleic acid binding |
| chr4D | 16813631 | 16819953 | + | *TraesCS4D02G039400* | ATP binding |
| chr4D | 16914928 | 16924027 | + | *TraesCS4D02G039500* | lipid binding |
| chr4D | 17248833 | 17253240 | - | *TraesCS4D02G039700* | phosphatidylinositol phosphate kinase activity |
| chr4D | 17859800 | 17865189 | - | *TraesCS4D02G040000* | ATP binding |
| chr4D | 18688930 | 18698103 | - | *TraesCS4D02G040200* | integral component of membrane |
| chr4D | 19177455 | 19188329 | - | *TraesCS4D02G040700* | integral component of chloroplast outer membrane |
| chr4D | 19290661 | 19292382 | + | *TraesCS4D02G040800* | phospholipid metabolic process |
| chr4D | 19407228 | 19410164 | - | *TraesCS4D02G041400* | ATP binding |
| chr4D | 19752189 | 19755893 | - | *TraesCS4D02G042300* | ATP binding |
| chr4D | 19774279 | 19784471 | + | *TraesCS4D02G042400* | ATP binding, protein phosphorylation |
| chr4D | 19900383 | 19900629 | + | *TraesCS4D02G042700* | proton-transporting ATP synthase complex, coupling factor F(o) |
| chr4D | 19901078 | 19902445 | + | *TraesCS4D02G042800* | chloroplast,ATP synthesis coupled proton transport |
| chr4D | 19903471 | 19904047 | + | *TraesCS4D02G042900* | ATP synthesis coupled with proton transport |
| chr4D | 19917552 | 19917684 | - | *TraesCS4D02G043200* | photosynthesis |
| chr4D | 19917787 | 19918009 | + | *TraesCS4D02G043300* | photosynthesis |
| chr4D | 19920421 | 19920944 | + | *TraesCS4D02G043400* | electron transporter, transferring electrons within the cyclic electron transport pathway of photosynthesis activity |
| chr4D | 19927824 | 19928925 | + | *TraesCS4D02G044000* | chloroplast |
| chr4D | 20405488 | 20409639 | + | *TraesCS4D02G044600* | ATP binding |
| chr4D | 20471924 | 20478349 | - | *TraesCS4D02G044900* | ATP binding |
| chr4D | 20897901 | 20899133 | + | *TraesCS4D02G045400* | photosynthesis, photosystem II oxygen evolving complex |
| chr4D | 21138832 | 21150226 | + | *TraesCS4D02G045500* | protein phosphorylation |
| chr4D | 21686214 | 21692418 | + | *TraesCS4D02G045900* | protein phosphorylation |
| chr4D | 21692429 | 21697046 | - | *TraesCS4D02G046000* | ATP hydrolysis activity |
| chr4D | 21748799 | 21770915 | + | *TraesCS4D02G046100* | phosphatidylinositol phosphate biosynthetic process |
| chr4D | 22233114 | 22236347 | - | *TraesCS4D02G046900* | protein phosphorylation |
| chr4D | 22938811 | 22945799 | + | *TraesCS4D02G047300* | carbohydrate metabolic process |
| chr4D | 22946577 | 22949866 | - | *TraesCS4D02G047400* | ligase activity |
| chr4D | 23316721 | 23323910 | + | *TraesCS4D02G047500* | pyridoxal phosphate binding |
| chr4D | 23412775 | 23419191 | + | *TraesCS4D02G047600* | GTP binding |
| chr4D | 23420023 | 23426081 | - | *TraesCS4D02G047800* | chloroplast, ATP synthesis coupled electron transport |
| chr4D | 23481632 | 23482682 | - | *TraesCS4D02G047900* | protein phosphorylation |
| chr4D | 25193487 | 25194492 | - | *TraesCS4D02G048800* | ATP binding |
| chr4D | 25484215 | 25490526 | + | *TraesCS4D02G049700* | pollen tube guidance |
| chr4D | 26244143 | 26250785 | + | *TraesCS4D02G050400* | phosphorylation |
| chr4D | 26920101 | 26929272 | - | *TraesCS4D02G051000* | integral component of membrane |
| chr4D | 28442341 | 28449763 | - | *TraesCS4D02G052100* | protein phosphorylation |
| chr4D | 28456336 | 28461004 | - | *TraesCS4D02G052200* | detection of visible light |
| chr4D | 28481268 | 28485238 | - | *TraesCS4D02G052400* | integral component of membrane |
| chr5A | 613539449 | 613543349 | + | *TraesCS5A02G429000* | ATP binding |
| chr5A | 613580722 | 613588536 | + | *TraesCS5A02G429300* | chloroplast |
| chr5A | 613699486 | 613702073 | + | *TraesCS5A02G429600* | carbohydrate metabolic process |
| chr5A | 614339665 | 614340136 | - | *TraesCS5A02G430400* | integral component of membrane |
| chr5A | 615312043 | 615318815 | + | *TraesCS5A02G431600* | ATP binding |
| chr5A | 616518366 | 616523495 | + | *TraesCS5A02G432000* | phosphatase activity |
| chr5A | 617183145 | 617186426 | + | *TraesCS5A02G433600* | carbohydrate binding |
| chr5A | 617251250 | 617255279 | - | *TraesCS5A02G434300* | protein phosphorylation |
| chr5A | 618949066 | 618954235 | - | *TraesCS5A02G435600* | integral component of membrane |
| chr5A | 618965641 | 618968705 | - | *TraesCS5A02G435800* | response to light intensity |
| chr5A | 619172815 | 619181657 | - | *TraesCS5A02G436300* | pyridoxal phosphate binding |
| chr5A | 619265485 | 619266034 | + | *TraesCS5A02G436600* | chloroplast |
| chr5A | 619273592 | 619273736 | - | *TraesCS5A02G437000* | photosynthesis |
| chr5A | 619273839 | 619273956 | - | *TraesCS5A02G437100* | chloroplast thylakoid membrane, chloroplast, photosynthesis |
| chr5A | 619682745 | 619683556 | - | *TraesCS5A02G437700* | integral component of membrane |
| chr5A | 619693367 | 619694776 | - | *TraesCS5A02G438000* | integral component of membrane |
| chr5A | 621746704 | 621759056 | + | *TraesCS5A02G440300* | GTP binding |
| chr5A | 621852720 | 621854882 | - | *TraesCS5A02G440600* | ADP binding |
| chr5A | 622767906 | 622773553 | - | *TraesCS5A02G442200* | ATP binding |
| chr5A | 622773719 | 622780594 | + | *TraesCS5A02G442300* | ATP binding |
| chr5A | 622780540 | 622783342 | - | *TraesCS5A02G442400* | acid phosphatase activity |
| chr5A | 622986184 | 622990751 | + | *TraesCS5A02G442500* | nucleic acid binding |
| chr5A | 623492643 | 623493736 | - | *TraesCS5A02G443300* | integral component of membrane |
| chr5A | 623552323 | 623556496 | - | *TraesCS5A02G443500* | GTPase activity |
| chr5A | 623957097 | 623957508 | - | *TraesCS5A02G444000* | nucleic acid binding |
| chr5A | 624209784 | 624212676 | - | *TraesCS5A02G444100* | integral component of membrane |
| chr5A | 624710649 | 624713923 | - | *TraesCS5A02G444600* | ATP binding |
| chr5A | 624722258 | 624725853 | - | *TraesCS5A02G444700* | ATP binding |
| chr5A | 624744062 | 624758677 | - | *TraesCS5A02G445000* | carbohydrate binding |
| chr5A | 624787299 | 624792782 | - | *TraesCS5A02G445100* | GTP binding |
| chr5A | 625732513 | 625738763 | - | *TraesCS5A02G445700* | ATP binding |
| chr5A | 626024210 | 626030298 | + | *TraesCS5A02G445900* | chloroplast |
| chr5A | 626059347 | 626067611 | + | *TraesCS5A02G446000* | nucleic acid binding |
| chr5A | 626285928 | 626288078 | - | *TraesCS5A02G446900* | ADP binding |
| chr5A | 626966025 | 626975963 | - | *TraesCS5A02G447200* | ATP binding |
| chr5A | 631051089 | 631060047 | + | *TraesCS5A02G448600* | nucleic acid binding |
| chr5A | 631329471 | 631330788 | + | *TraesCS5A02G449000* | ATP binding |
| chr5A | 631503085 | 631505615 | + | *TraesCS5A02G449400* | ATP binding |
| chr5A | 631507174 | 631513881 | - | *TraesCS5A02G449500* | integral component of membrane |
| chr5A | 632356856 | 632359157 | + | *TraesCS5A02G450600* | integral component of membrane |
| chr5A | 632835355 | 632837574 | + | *TraesCS5A02G450900* | protein phosphorylation |
| chr5A | 634085971 | 634088352 | + | *TraesCS5A02G452700* | integral component of membrane |
| chr5A | 634357112 | 634358466 | - | *TraesCS5A02G452800* | phosphorelay signal transduction system |
| chr5A | 635245023 | 635246377 | + | *TraesCS5A02G453600* | phosphorelay signal transduction system |
| chr5A | 635407573 | 635408909 | - | *TraesCS5A02G454200* | chloroplast thylakoid membrane, chlorophyll binding |
| chr5A | 635410443 | 635411662 | - | *TraesCS5A02G454300* | chloroplast thylakoid membrane, chlorophyll binding |
| chr5A | 635414686 | 635415387 | - | *TraesCS5A02G454400* | chloroplast thylakoid membrane, chlorophyll binding |
| chr5A | 635504405 | 635509559 | - | *TraesCS5A02G454600* | ATP binding |
| chr5A | 635960776 | 635965228 | + | *TraesCS5A02G455700* | integral component of membrane |
| chr5A | 636305515 | 636308414 | - | *TraesCS5A02G455800* | chlorophyllide a oxygenase activity |
| chr5A | 636411561 | 636414021 | + | *TraesCS5A02G456000* | chlorophyllide an oxygenase activity |
| chr5A | 636925082 | 636926961 | + | *TraesCS5A02G457000* | GTP binding |
| chr5A | 636928041 | 636930556 | + | *TraesCS5A02G457100* | GTPase activity |
| chr5A | 637167477 | 637168439 | + | *TraesCS5A02G457500* | photosynthesis |
| chr5A | 638068421 | 638069918 | - | *TraesCS5A02G457900* | ATP hydrolysis activity |
| chr5A | 638381179 | 638382537 | + | *TraesCS5A02G458200* | phosphorelay signal transduction system |
| chr5A | 638817814 | 638819092 | + | *TraesCS5A02G458600* | protein phosphorylation |
| chr5A | 639306149 | 639307856 | - | *TraesCS5A02G459200* | chloroplast stroma, photosynthesis |
| chr5A | 640724646 | 640726263 | + | *TraesCS5A02G460300* | inorganic phosphate transmembrane transporter activity |
| chr5A | 640728883 | 640733265 | - | *TraesCS5A02G460400* | phosphatase activity |
| chr5A | 641153773 | 641158776 | + | *TraesCS5A02G460900* | RNA binding |
| chr5A | 641220051 | 641222381 | - | *TraesCS5A02G461000* | ATP binding |
| chr5A | 641723797 | 641726781 | + | *TraesCS5A02G461300* | acid phosphatase activity |
| chr5A | 641732244 | 641734013 | + | *TraesCS5A02G461500* | photosynthesis |
| chr5A | 643328442 | 643333346 | - | *TraesCS5A02G463100* | protein phosphorylation |
| chr5A | 643357091 | 643359406 | - | *TraesCS5A02G463200* | protein phosphorylation |
| chr5A | 643363992 | 643366230 | - | *TraesCS5A02G463300* | protein phosphorylation |
| chr5A | 643924797 | 643926446 | - | *TraesCS5A02G464500* | carbohydrate metabolic process |
| chr5A | 644123518 | 644129496 | - | *TraesCS5A02G465000* | GTPase activity |
| chr5A | 644436547 | 644439401 | + | *TraesCS5A02G465700* | protein phosphorylation |
| chr5A | 644805441 | 644812565 | + | *TraesCS5A02G466100* | chlorophyll biosynthetic process, photosynthesis |
| chr6A | 62130060 | 62130723 | + | *TraesCS6A02G095500* | integral component of membrane |
| chr6A | 63116735 | 63120385 | + | *TraesCS6A02G096000* | nucleic acid binding |
| chr6A | 64255216 | 64262082 | + | *TraesCS6A02G097000* | integral component of membrane |
| chr6A | 65434223 | 65437470 | + | *TraesCS6A02G098300* | lipid metabolic process |
| chr6A | 65681324 | 65684847 | - | *TraesCS6A02G098500* | integral component of membrane |
| chr6A | 67126389 | 67137086 | - | *TraesCS6A02G099600* | ATP binding |
| chr6A | 67374165 | 67378249 | - | *TraesCS6A02G099900* | protein phosphorylation |
| chr6A | 67494390 | 67496461 | + | *TraesCS6A02G100100* | nucleic acid binding |
| chr6A | 67498142 | 67499802 | + | *TraesCS6A02G100200* | nucleic acid binding |
| chr6A | 68546445 | 68549501 | + | *TraesCS6A02G100900* | ATP hydrolysis activity |
| chr6A | 69137383 | 69139546 | - | *TraesCS6A02G101200* | GTP binding |
